# Foam-Enhanced Air Sparging Coupled with Soil Vapor Extraction for Remediation of Trichloroethylene DNAPL-Contaminated Soil

**DOI:** 10.1021/acsomega.6c03103

**Published:** 2026-08-29

**Authors:** Xuyen Thi Hong Luong, Chenju Liang

**Affiliations:** Department of Environmental Engineering, 34916 National Chung Hsing University, 145 Xingda Rd., South Dist., Taichung 402, Taiwan

## Abstract

Conventional remediation technologies, such as soil vapor extraction (SVE) and air sparging combined with SVE (AS-SVE), are often constrained by gas channeling and mass-transfer limitations when treating trichloroethylene (TCE) dense nonaqueous phase liquid (DNAPL) source zones. To overcome these challenges, this study developed an integrated oxidative foam-enhanced AS-SVE (Oxi-FEAS-SVE) system to improve contaminant accessibility, contaminant mass transfer, and degradation efficiency. The system employed oxidative foam composed of 32 mM α-olefin sulfonate (AOS) and 1500 mM sodium persulfate (SPS) to replace conventional air injection, with an AS/SVE flow ratio of 1:1 in a 5 L cylindrical soil reactor. Under these conditions, SVE alone and AS-SVE removed less than 2% of TCE through volatilization, whereas the Oxi-FEAS-SVE system achieved a total removal efficiency of 91% in this laboratory-scale study. This enhanced performance resulted from a two-stage process: an initial 3 h active operation that removed 54% of TCE primarily through DNAPL volatilization, followed by a 48 h static period that contributed an additional 37% removal through oxidative mineralization. Mechanistically, the AOS/SPS oxidative foam performs dual functions. It enhances interfacial contact area, promoting dissolution and volatilization for SVE capture, while simultaneously generating sulfate radicals via ambient-temperature activation of SPS to sustain oxidative degradation. Detected intermediates (C_2_H_2_Cl_4_, CHCl_3_, CH_2_Cl_2_, and CH_3_Cl) suggest degradation pathways involving radical addition, hydrogen abstraction/addition, and sequential dechlorination. Overall, the Oxi-FEAS-SVE system demonstrates promise as an innovative approach for treating TCE DNAPL source zones, while highlighting the need for additional studies to evaluate its viability under variable site conditions.

## Graphical abstract

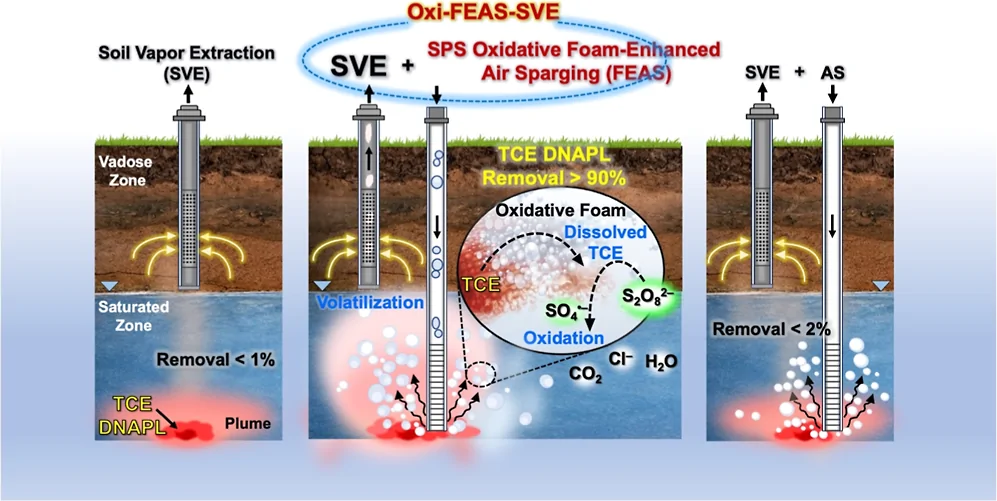

## 1 Introduction

Trichloroethylene (TCE, C_2_HCl_3_) is a widely used volatile organic compound (VOC) and one of the most common groundwater contaminants worldwide. Its historical use as a metal degreaser, dry-cleaning solvent, and chemical intermediate has resulted in extensive soil and groundwater pollution.^1,2^ As of 2017, TCE has been identified at over 56% of the 1854 hazardous waste sites included on the United States Environmental Protection Agency (USEPA) National Priorities List.^3^ Remediation of TCE is particularly challenging due to its behavior as a dense nonaqueous phase liquid (DNAPL). Being denser than water and poorly miscible, TCE migrates downward through the subsurface until encountering low-permeability layers, where it accumulates as DNAPL pools at the base of aquifers.^4,5^ These pools serve as long-term contamination sources, slowly releasing dissolved TCE into groundwater for decades and limiting the effectiveness of conventional pump-and-treat (P&T).^6^ Among in situ technologies, air sparging (AS) is also widely used to enhance removal through volatilization and aerobic biodegradation of VOC contaminants.^7,8^

The effectiveness of AS strongly depends on the phase of the contaminant. While AS can effectively remove dissolved TCE in the aqueous phase, injecting air directly into pure-phase DNAPL does not significantly enhance volatilization, and removal is therefore largely limited to slow biodegradation. In typical applications, AS wells are installed beneath a widespread dissolved plume,^7^ whereas effective DNAPL treatment requires injection points to be positioned as close as possible to the DNAPL pool.^9^ Even with optimal placement, remediation is constrained by severe mass-transfer limitations due to the minimal contact area between injected air and the immiscible TCE liquid. Enhancing DNAPL dissolution into the aqueous phase is therefore critical to increase contaminant availability for air–water interaction. In this context, surfactant foam has emerged as an efficient alternative to conventional air injection for improving DNAPL accessibility and mass transfer. Maire et al.^10^ demonstrated that foam injection at the AS point enhances DNAPL dissolution and recovery by producing a more homogeneous dissolved plume. Foam also increases the interfacial surface area between air bubbles and TCE, thereby accelerating mass transfer from the DNAPL phase to the vapor phase.^11–14^ By utilizing only minimal surfactant, foam injection minimizes the risks of unintended contaminant migration associated with conventional high-volume surfactant flushing. Consequently, replacing air with foam in existing AS-SVE systems can increase TCE volatilization rates and SVE capture efficiency, offering a potentially more effective and controlled remediation approach.

Furthermore, the integration of surfactant foam with in situ chemical oxidation (ISCO) using persulfate (S_2_O_8_^2–^, PS) has led to the development of oxidative foams that provide a dual-action mechanism of contaminant solubilization and chemical degradation.^15^ Bajagain et al.^16^ investigated a PS-bioaugmentation foam system for the remediation of soil total petroleum hydrocarbons (TPHs) and reported that foam application enhanced soil infiltration and microbial activity. Their results showed approximately 80% TPH degradation, compared with only 52% removal achieved by bioaugmentation alone. Building on this concept, Luong and Liang^15^ developed a PS-based oxidative foam composed of sodium α-olefin sulfonate (AOS, 32 mM) and sodium persulfate (SPS, 50 mM or 1700 mM), generating a high-quality foam (>99% gas phase, <1% surfactant solution). When applied to treat approximately 10 g of TCE DNAPL in 1 L of aqueous solution, the AOS/PS oxidative foam dissolved 60–80% of TCE within 2 h. This performance represented nearly a 10-fold increase in dissolution efficiency compared with nitrogen (N_2_) sparging or liquid AOS surfactant treatments under similar conditions. The enhancement was attributed to surfactant-stabilized interfaces within the foam lamellae, which promoted TCE adsorption and increased its partitioning into gas bubbles, thereby accelerating volatilization. Simultaneously, PS in the liquid phase enabled chemical oxidation, achieving 40–74% TCE mineralization rather than mere physical dissolution observed with conventional surfactant foams. These findings demonstrated the superior performance of oxidative foam compared with conventional air sparging. However, translating this approach to field applications requires evaluation under more realistic subsurface conditions. Based on the enhanced dissolution and oxidation potential observed in aqueous systems,^15^ the AOS/SPS oxidative foam formulation was selected for further investigation in this study. Accordingly, this work expanded the oxidative foam-enhanced air sparging (Oxi-FEAS) concept by integrating it with SVE to form an Oxi-FEAS-SVE system for the remediation of TCE DNAPL in silica sand porous media, simulating aquifer conditions. In addition, the formation of reaction byproducts was examined to elucidate TCE degradation pathways and provide a comprehensive evaluation of the Oxi-FEAS-SVE treatment process.

## 2 Materials and Methods

### 2.1 Chemicals and Materials

Water was prepared using reverse osmosis (RO) purification system (XL-300A, Sky Water Ltd. Taiwan), and had a pH of approximately 6.8 and a conductivity of approximately 5.0 μs cm^–1^. Sodium α-olefin sulfonate (C_14_–C_16_, ≥97.0%) was purchased from King Yu Chemicals Co., Ltd. (Taiwan). Trichloroethylene (C_2_HCl_3_, ≥99.9%) was purchased from Sigma-Aldrich (St. Louis, MO, USA). Sodium persulfate (Na_2_S_2_O_8_, ≥99.0%) was purchased from Merck (Germany). Methanol (CH_3_OH, >99.9%) was purchased from Echo Chemical Co., Ltd. (Taiwan). *n*-Hexane (C_6_H_14_, ≥95.0%) was purchased from Tedia Chemicals, USA. Filtrasorb 400 (F400) granular activated carbon (GAC) was purchased from Li Jing Viscard Co., Ltd. (Taiwan). Acetonitrile (C_2_H_3_N, >99.9%) was purchased from Aencore Chemical PTY., Ltd. (Australia). Potassium iodide (KI, ≥99.7%), sodium bicarbonate (NaHCO_3_, >99.6%), and sodium nitrate (NaNO_3_, ≥99%) were purchased from Union Chemical Works (Taiwan). Sodium chloride (NaCl, ≥99.5%) was purchased from Honeywell (Germany). Silica sand (SiO_2_) was purchased from Yow Shiuan Co., Ltd. (Taiwan), with particle size mesh #30–70. Bentonite was purchased from First Chemical Works, Inc. (Taiwan).

### 2.2 Experimental Procedure

The schematic and actual experimental setup of the Oxi-FEAS-SVE system used in this study are shown in Figure 1a,b, respectively. To simulate subsurface conditions, a 5 L cylindrical glass reactor (18 cm diameter, 25 cm height) was packed with 9200 g of silica sand, forming a layered soil profile consisting of a 5 cm saturated zone at the bottom, an approximate 3 cm capillary fringe, and a 15 cm unsaturated zone. A 2 cm bentonite layer was placed on top of the unsaturated zone (Figure 1c) to simulate an impermeable paved surface and prevent TCE vapor from escaping into the atmosphere. The physical properties of the silica sand and bentonite, as well as reactor specifications, are summarized in Table S1 in the Supporting Information. It should be noted that inert silica sand was used as the porous medium in this study; therefore, the natural oxygen demand (NOD) was considered negligible for this foundational assessment. However, NOD is recognized as a significant factor influencing oxidant consumption and treatment performance during field-scale ISCO applications. Accordingly, site-specific NOD evaluation should be conducted in future applications involving natural aquifer materials or field-contaminated soils. To represent a TCE DNAPL source zone, 4.2 mL of TCE DNAPL (∼6132 mg) was injected at the bottom of the saturated zone. This dosage corresponds to more than 10 times the theoretical mass required to generate the maximum saturated TCE vapor concentration (∼410 mg L^–1^) in the unsaturated zone. Establishing this excess DNAPL created a complex contamination environment containing both residual and pooled DNAPL, thereby providing a stringent condition for evaluating the performance of the Oxi-FEAS-SVE system under DNAPL source-zone conditions. A N_2_/foam sparging injection well was installed at the center of the saturated zone and positioned as close as possible to the DNAPL pool (Figure 1d). Foam was generated following the procedure established by Luong and Liang (2025).^15^ Briefly, foam was produced at a fixed liquid-to-gas ratio by continuously delivering the AOS surfactant solution using a peristaltic pump, while N_2_ gas was introduced into a 2 L AOS surfactant solution at a flow rate of 2 L min^–1^. During foam generation and injection, the internal pressure of the foam generator was monitored and controlled below 1.5 kg cm^–2^ using pressure regulation and release valves. The resulting AOS foam had a foam quality of 99.6%, indicating a gas-dominated flow system in which the gas phase primarily governs the overall flow behavior. Therefore, conventional liquid-phase rheological characterization was not prioritized in this assessment.

**Figure 1:**
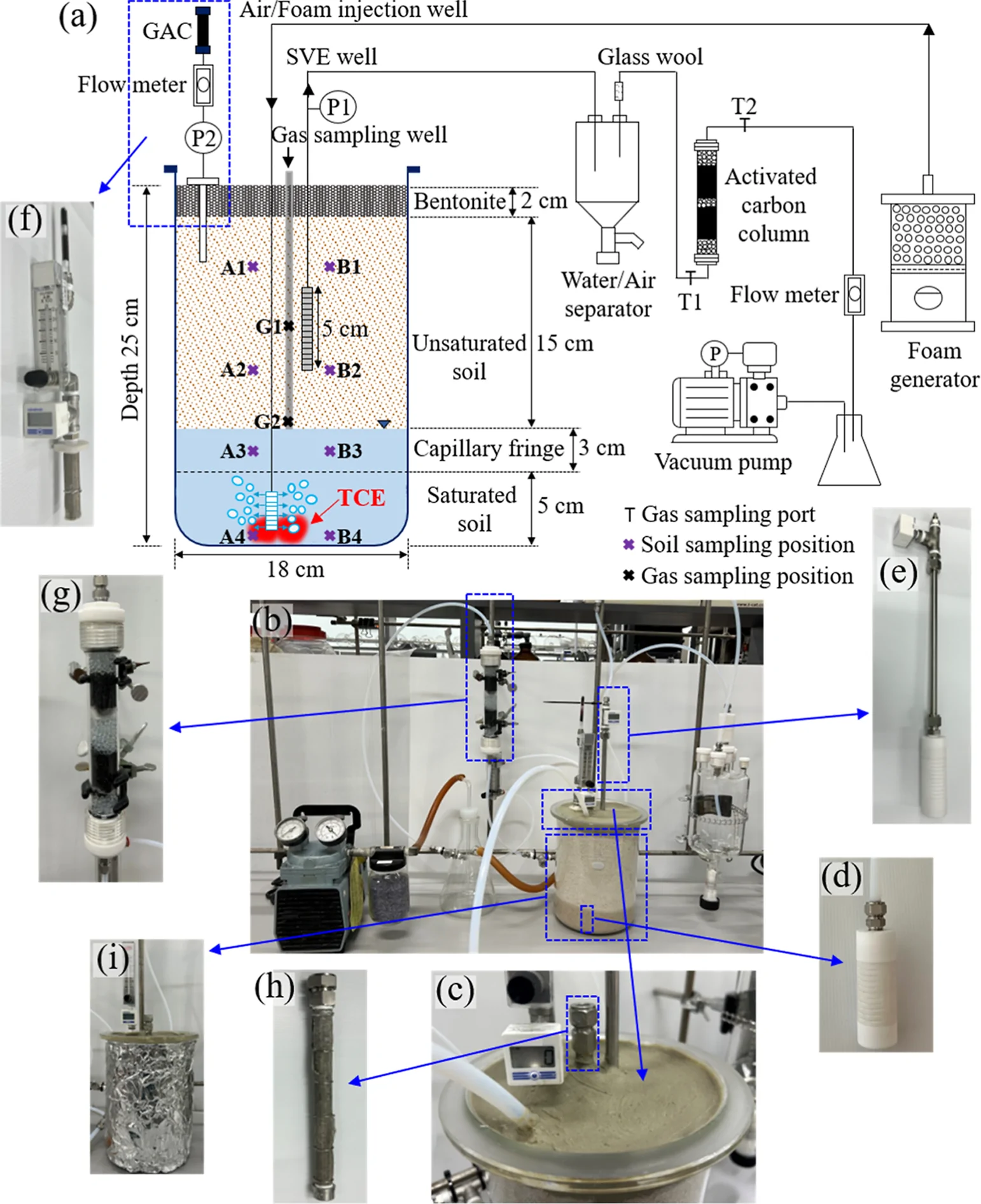
Oxi-FEAS-SVE experimental setup and components: (a) schematic diagram of the system; (b) photograph of the setup; (c) top bentonite sealing layer; (d) air sparging well; (e) soil vapor extraction well; (f) monitoring well equipped with a digital pressure gauge and flow meter connected to an Orbo tube; (g) granular activated carbon column; (h) gas sampling well; and (i) aluminum foil-wrapped reactor.

A SVE well was installed in the unsaturated zone with a screen length of 5 cm to extract soil vapor. A vacuum pump connected to the outlet was used to operate the SVE system, while a flow meter monitored the gas flow rate. To ensure effective removal of volatilized contaminants, the SVE extraction flow rate (*Q*_SVE_) was calculated using the following eq 1 ^17^

1 $$Q_{SVE}=\frac{PVER\times \pi \times r^{2}\times h\times n_{e}}{t}$$

where PVER represents the pore volume exchange rate; *r* (cm) is the radius of well influence over which the minimum pore volume exchange is desired; *h* (cm) is the thickness of unsaturated soil layer; *n*_e_ is the effective soil porosity; and *t* (min) is the operating time.

The extraction flow rate *Q*_SVE_ was modeled as a function of the radius of influence (ROI, 0–9 cm) and varying PVER values (1–30), with the results summarized in Table S2 (Supporting Information). To ensure effective vapor capture and allow a rigorous evaluation of TCE mass transfer, a high-intensity extraction strategy was adopted. Specifically, an ROI of 9 cm, corresponding to the full reactor radius, and a PVER of 30 were selected. Based on eq 1, these conditions yielded a calculated *Q*_SVE_ of 250 mL min^–1^. The system was operated for 3 h to provide sufficient residence time for volatilized TCE recovery while maintaining a stable vapor exchange rate within the laboratory-scale reactor. To maintain geotechnical stability and reduce the risk of soil fracturing during Oxi-FEAS application, the experimental design emphasized low-pressure operation. A balanced 1:1 injection-to-extraction ratio (*Q*_AS_/*Q*_SVE_ = 1:1) was applied throughout the experiment to prevent excessive pressure buildup within the reactor. This operating condition helped maintain a stable and contained pressure field within the soil matrix, thereby reducing lateral pressure gradients that could promote short-circuiting along the reactor walls. Pressure containment was verified using internal pressure gauges P1 and P2. The pressure measured near the reactor boundary at P2 (*r* = 9 cm) remained at atmospheric pressure (0.00 psi) throughout the experiments. These results indicate that the system effectively minimized overpressure while confining the treatment influence within the soil matrix, thereby reducing boundary-induced flow bias and supporting the 9 cm ROI as the effective treatment zone. Gauge P1 was installed directly at the SVE well to measure the vacuum pressure generated during the extraction process (Figure 1e). A second monitoring point (P2) was established at a radial distance of 9 cm from the SVE well to observe pressure propagation within the porous medium. This location was also equipped with a flow meter and a charcoal Orbo tube (ORBO-32, USA) for vapor sampling (Figure 1f). Monitoring pressure and gas flow at these two locations enabled evaluation of the pressure distribution and subsurface response to combined air/foam injection and SVE operation. It should be noted that the 5 L reactor was designed to provide a controlled proof-of-concept evaluation of the proposed FEAS remediation approach. However, the relatively small reactor scale and limited saturated zone of 5 cm differ from field conditions, where DNAPL source zones may occur within thicker saturated intervals and more heterogeneous subsurface formations. During AS and SVE, the limited saturated zone in the laboratory reactor may have become partially or largely desaturated more rapidly than would be expected in many field settings. Therefore, the results obtained from this setup should be interpreted as demonstrating the feasibility of the Oxi-FEAS-SVE concept under controlled laboratory conditions, rather than as directly representing field-scale performance. Scaling this technology to field applications may involve additional challenges, including subsurface heterogeneity, pressure loss during injection and extraction, foam stability under site-specific geological conditions, and the need to maintain effective vapor capture. This laboratory setup thus serves as a foundational step toward future pilot- or field-scale investigations.

The extracted gas was first passed through a water–air separator and glass wool to remove entrained moisture, and subsequently directed to a GAC column (30 cm length, 2.54 cm I.D.), where volatilized TCE was adsorbed during the SVE process. Remediation performance was evaluated by measuring TCE concentrations in soil samples collected from positions A1–A4 and B1–B4 (Figure 1a), as well as in the extracted vapor stream passing through the GAC column (Figure 1g). During operation, gas samples were periodically collected from four designated sampling points: G1 (9.5 cm below ground surface, bgs) and G2 (17 cm bgs) in the gas sampling well (Figure 1h), and T1 and T2 located before and after the GAC column, respectively. The experimental design and operating conditions for the four treatment groups, control, SVE alone, AS-SVE, and Oxi-FEAS-SVE (including regular and oxidative foam), are summarized in Table S3 (Supporting Information). To eliminate potential photodegradation of TCE by laboratory lighting, the reactor was fully wrapped (Figure 1i) throughout the experiments. Consequently, the observed TCE removal and byproduct formation can be attributed primarily to the combined effects of foam injection (regular or oxidative) and vacuum extraction. Each experimental group listed in Table S3 (Supporting Information) was conducted as an independent reactor test using freshly packed silica sand and newly introduced TCE contamination. The treatments were not performed sequentially in the same reactor. All experiments were performed in triplicate, and the results are reported as average values with one standard deviation.

### 2.3 Analytical Methods

TCE in soil samples and GAC-sorbed TCE were extracted using hexane prior to analysis. Briefly, 30 g of soil or 25 g of GAC were transferred into 30 mL amber screw-cap glass bottles fitted with polytetrafluoroethylene (PTFE)/silicone septa. After adding 10 mL of hexane, the samples were shaken at 1500 rpm for 2 min, allowed to rest for 1 min, and shaken again for an additional 2 min. The mixtures were then centrifuged at 1000 rpm for 5 min. A 2 mL aliquot of the supernatant was collected and filtered through a 0.2 μm PTFE membrane using a stainless-steel syringe holder (Advantec, KS-13). The filtered extract was subsequently analyzed by high-performance liquid chromatography (HPLC; PerkinElmer Flexar LC system) equipped with a photodiode array (PDA) detector. HPLC/PDA operating conditions followed the method reported by Luong and Liang.^15^

For gaseous analysis, TCE samples were collected using a gastight syringe equipped with a 25 cm needle (VICI gas syringe series A-2, USA) and analyzed by gas chromatography (GC, Agilent 7890A) coupled with mass spectrometry in electron impact mode (MS, Agilent 5975C). Compound separation was performed using an Agilent J&W Scientific DB-624 column. TCE degradation byproducts were analyzed using a purge-and-trap system (O.I. Analytical Eclipse 4760) followed by qualitative and quantitative GC/MS analysis in accordance with NIEA Method W785.54B. This method was used to screen for 60 potential volatile organic compounds (VOCs), including vinyl chloride. In addition, SPS concentrations were determined at 400 nm using a UV–vis spectrophotometer (HACH DR/3900),^18^ while chloride ion concentrations were measured using an ion-selective electrode (ISE; Orion 9617B) connected to a dual-channel benchtop meter (HANNA HI 5222).

## 3 Results and Discussion

### 3.1 Assessment of the Synergistic Remediation Efficiency of Integrated Oxi-FEAS-SVE for TCE DNAPL Contamination

The relationship between *Q*_SVE_ and vacuum pressure (*S*) distribution was evaluated at the SVE extraction well (P1) and at an observation point (P2, *r* = 9 cm), as summarized in Table S4 (Supporting Information). At the extraction well (P1), vacuum pressure increased markedly with increased flow rate, ranging from −0.15 psi at 100 mL min^–1^ to −9.86 psi at 1200 mL min^–1^. This trend indicates a strong dependence of the required driving force on the extraction rate needed to overcome the pneumatic resistance of the silica sand medium. In contrast, the vacuum pressure measured at the observation point (P2) remained at 0.00 psi for all tested flow rates, which is below the detection limit of the digital pressure gauge (0.03 psi). The measured values were subsequently compared with theoretical predictions derived from the Hantush leaky equation (eq 2)^19,20^

2 $$S_{P2}=\frac{{3.3Q}_{SVE}}{T}\times K_{0}\left[\frac{r}{B}\right]$$

where *S*_P2_ (inches of H_2_O) is the air vacuum measured at observation point P2; *r* (ft) is the radial distance from the SVE well to the observation point; *B* is the leaky factor, defined as 0.89 × radius of influence; *K*_0_ is the zero-order modified Bessel function of the second kind; and *T* (ft^2^ day^–1^) is gas transmissivity. The value of *T* is calculated using eq 3, where *b* (ft) is the unsaturated zone thickness (15 cm) and *K*_A_ (ft day^–1^) is the air permeability obtained from eq 4

3 $$T=b\times K_{A}$$

4 $$K_{A}=\frac{K_{W}\mu_{W}\rho_{A}}{\rho_{W}\mu_{A}}$$

where *K*_W_ (ft day^–1^) represents the hydraulic conductivity of water, μ_W_ and μ_A_ (N day ft^–2^) denote the viscosities of water and air, respectively; and ρ_W_ and ρ_A_ (kg ft^–3^) represent the densities of water and air, respectively.

Theoretical calculations for *S*_P2_ yielded negligible vacuum pressures ranging from −1.1 × 10^–4^ to −12.9 × 10^–4^ psi (Table S4 (Supporting Information)). The measured pressure at P2 remained effectively atmospheric (0.00 psi), consistent with these predictions. Given that the pressure gauge at P2 has a detection limit of 0.03 psi, the calculated pressures fall well below the instrument’s resolution, explaining why the monitored value appeared as 0.00 psi. These results indicate that, at the applied flow rates, the ROI did not extend to the 9 cm boundary. In this configuration, the AS well is located in the saturated zone while the SVE well is positioned directly above it in the unsaturated zone. A balanced injection–extraction ratio (1:1) ensures that the volume of gas or foam injected at the base is matched by the extraction rate at the top. This balance prevents subsurface pressure buildup that could otherwise drive TCE vapors laterally toward the reactor boundary. The pressure data further confirm the suitability of this configuration. A significant vacuum measured at P1 creates a localized extraction zone, whereas the atmospheric pressure observed at the 9 cm observation point (P2) indicates that the vacuum influence does not propagate toward the reactor walls. Together, these conditions establish a strong vertical pressure gradient that directs TCE vapors and oxidative foam from the saturated zone upward into the unsaturated zone, where they are immediately captured by the SVE system. This configuration ensures centralized recovery of mobilized TCE and prevents fugitive vapor accumulation along the reactor perimeter.

TCE concentrations in the saturated and unsaturated zones were determined by soil sampling, with raw analytical data provided in Table S5 (Supporting Information) and spatial distributions illustrated in Figure 2. In the control experiment (Figure 2a), TCE remained concentrated at the base of the saturated zone, with an average concentration of 2814.9 ± 30.9 mg kg^–1^ dry soil at A4 and B4 after 3 h under static conditions (see Tables 1 and S5 (Supporting Information) for raw data). As shown in Figure 2b, SVE alone was ineffective for DNAPL recovery, with an average concentration of 2508.9 ± 26.9 mg kg^–1^ dry soil at the base of the saturated zone. Although TCE concentrations increased slightly in the capillary zone near the water table, rising from 65.3 ± 0.3 to 324.6 ± 11.0 mg kg^–1^ dry soil, source-zone concentrations decreased by only approximately 10% (Table 1). These findings indicate that SVE alone cannot effectively remediate submerged TCE DNAPL due to strong mass-transfer limitations.

**Figure 2:**
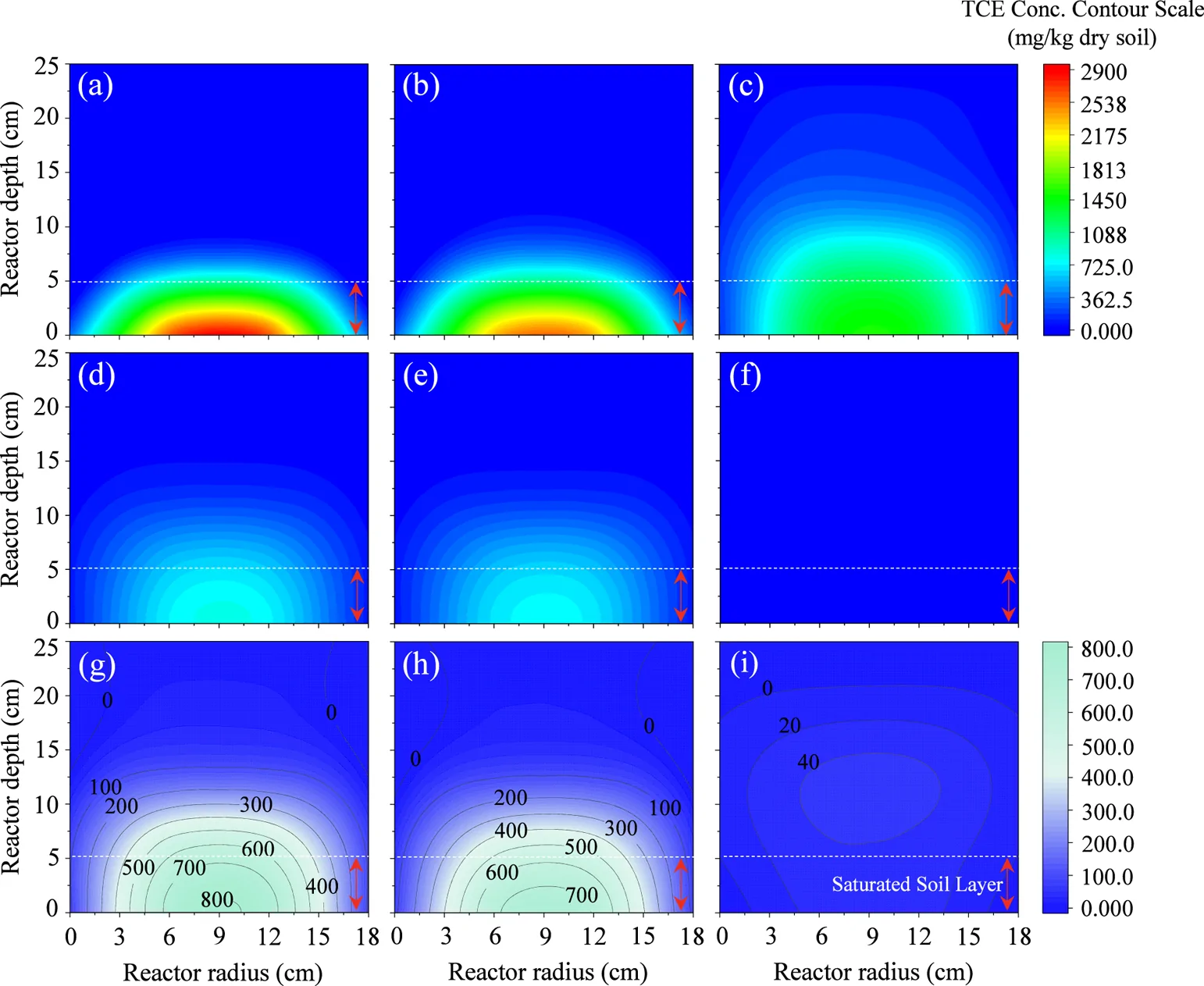
Contour maps of soil TCE concentrations in the reactor under different experimental conditions: (a) control (no treatment), (b) SVE alone, (c) air sparging coupled with SVE (AS-SVE), (d) foam-enhanced AS-SVE (FEAS-SVE) using AOS foam, (e) oxidative FEAS-SVE (Oxi-FEAS-SVE) using AOS/SPS foam after 3 h of operation, and (f) Oxi-FEAS-SVE after 3 h of operation followed by 48 h of static reaction. Panels (g–i) present enlarged views of the reduced concentration scales for conditions (d–f), respectively.

**Table 1:** Statistical Summary of Soil TCE Concentrations under Different Experimental Conditions^a^

	TCE concentration (mg kg^–1^ dry soil)		
	Layers		
Experiment	Unsaturated	Capillary rise	Saturated
(a)	7.8 ± 0.3	65.3 ± 0.3	2814.9 ± 30.9
(b)	49.1 ± 1.4	324.6 ± 11.0	2508.9 ± 26.9
(c)	232.0 ± 4.0	1076.0 ± 13.7	1481.0 ± 13.6
(d)	68.2 ± 0.4	599.9 ± 4.0	781.1 ± 6.2
(e)	53.5 ± 0.2	480.1 ± 5.5	730.3 ± 6.6
(f)	42.5 ± 0.5	43.8 ± 0.4	42.9 ± 0.4

a (a) Control experiment (no treatment), (b) SVE alone, (c) AS coupled with SVE (AS-SVE), (d) foam-enhanced AS-SVE (FEAS-SVE) using AOS foam, (e) oxidative FEAS-SVE (Oxi-FEAS-SVE) using AOS/SPS foam after 3 h of operation, and (f) Oxi-FEAS-SVE after 3 h of operation followed by 48 h of static reaction.

The integration of AS and SVE, operated at an N_2_ flow rate of 250 mL min^–1^ for 3 h, significantly enhanced TCE mobilization. Based on Table S5 (Supporting Information), approximately 35% of the initial TCE mass migrated from the saturated zone to the capillary and unsaturated zones. Note that N_2_ was utilized instead of O_2_, as reported by Teng et al. (2025),^21^ to maintain an oxygen-limited environment and to preclude aerobic microbial activity and potential biological degradation pathways during physical stripping. Compared with Figure 2a,b, increased TCE vertical transport demonstrates that the central SVE vacuum provides the driving force needed to overcome hydrostatic resistance in the saturated layer.

Despite the enhanced contaminant migration, Figure 2c shows the formation of a DNAPL smear zone trapped within the capillary fringe. At sampling locations A3 and B3, TCE concentrations remained high, averaging 1076.0 ± 13.7 mg kg^–1^ dry soil. This result indicates that gas channeling restricted the transport of volatilized TCE to the extraction well. This preferential flow behavior indicates that although the AS-SVE system improves contaminant extraction compared with SVE alone, inherent N_2_ gas channeling remains a major limitation, restricting effective transport of volatilized TCE to the extraction well. The mass distribution analysis in Figure 3 further confirms that the control, SVE-only, and conventional AS-SVE experiments (Figure 3a–c) exhibited minimal remediation performance, with more than 92% of the initial TCE mass persisting as soil residuals. These results demonstrate that discrete gas-phase stripping is insufficient to access sequestered DNAPL pools. To overcome these limitations, the study adopted a foam-enhanced approach. By introducing a small liquid fraction (<1% by volume), the foam functions as a contact-enhancement agent that increases the interfacial contact area between the treatment reagent and the soil matrix. In addition, by occupying pore spaces more effectively than discrete gas bubbles, the foam promotes a more uniform sweep of the saturated zone. This mechanism enables the surfactant solution to reach the DNAPL interface directly, thereby overcoming the limitations associated with preferential gas flow.

**Figure 3:**
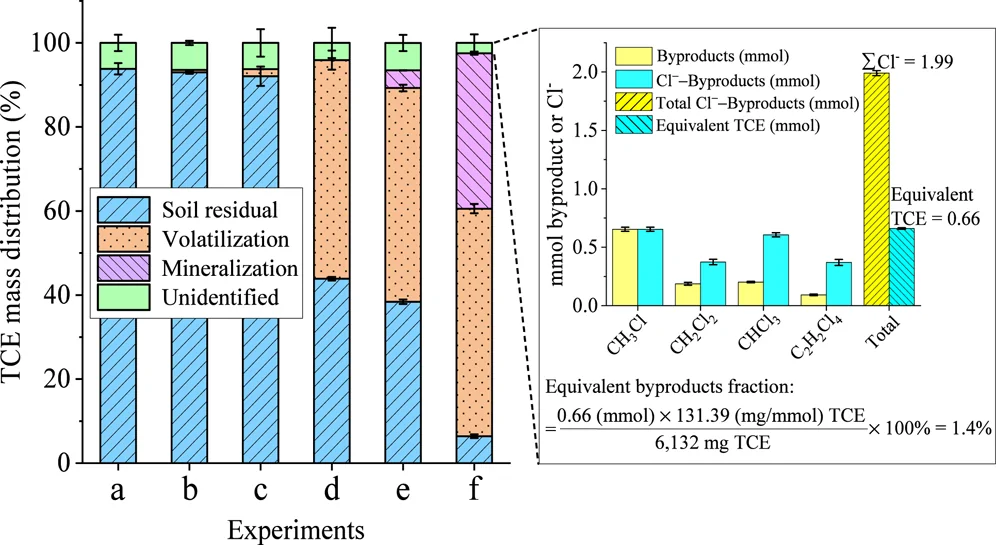
TCE mass distribution under different experimental conditions: control (no treatment, Experiment a); SVE alone (Experiment b); AS-SVE (Experiment c); FEAS-SVE utilizing AOS foam (Experiment d); Oxi-FEAS-SVE utilizing AOS/SPS foam after 3 h of operation (Experiment e); and Oxi-FEAS-SVE utilizing AOS/SPS foam after 3 h of operation followed by 48 h of static condition (Experiment f).

The selection of 32 mM AOS as the foaming agent in this study was based on findings reported by Luong and Liang,^15^ who evaluated several surfactants, polyoxyethylene (20) sorbitan monooleate (TW80), sodium dodecyl sulfate (SDS), AOS, and their mixtures, for foam stability and quality. Their results showed that AOS at 32 mM exhibited the highest foam stability (∼345 min) and superior foam quality (∼99.6%). Using this optimized formulation, the present study ensures that the generated foam remains stable as it migrates across the saturated–unsaturated interface. Such structural stability promotes more uniform subsurface sweep and enhances interfacial contact between the AOS/SPS foam and TCE, thereby improving contaminant dissolution and mineralization efficiency.

As shown in Figure 2d, the application of AOS foam resulted in a pronounced reduction of TCE across the soil profile. To better resolve the distribution of low residual concentrations, Figure 2g uses a refined concentration scale (0–800 mg kg^–1^ dry soil). This higher-resolution representation clearly indicates that residual TCE DNAPL mass was substantially reduced after a 3 h operational period. These results demonstrate that the integrated FEAS-SVE system achieved significantly greater dissolution and mass transfer of TCE compared with conventional AS-SVE, thereby facilitating more efficient contaminant transfer to the SVE well. The enhanced performance is further quantified by the mass distribution analysis shown in Figure 3. The FAES-SVE process increased TCE volatilization to approximately 50–52%, while reducing residual soil concentrations to about 44%. The FEAS-SVE system demonstrated enhanced remediation performance, reducing the average soil TCE concentration across the profile to 781.1 ± 6.2 mg kg^–1^ dry soil (Experiment d, Table 1). This represents a substantial decrease compared with the control experiment (Experiment a, 2814.9 ± 30.9 mg kg^–1^ dry soil) and the SVE-only experiment (Experiment b, 2508.9 ± 26.9 mg kg^–1^ dry soil), highlighting the improved mass-transfer capability of this system.

The superior performance of the foam-enhanced system can be attributed to the synergistic mechanisms introduced by AOS foam. Acting as a solubilization-enhancing reagent, the foam disrupts DNAPL pools and promotes dissolution of TCE previously trapped by capillary forces. This effect is driven by the unique structure of foam bubbles, which are surrounded by a thin surfactant-rich liquid layer known as the lamella.^22^ The lamella provides a large interfacial area where TCE molecules can accumulate and partition into the gas phase within the bubbles, thereby promoting rapid dissolution and volatilization. By distributing the stripping gas through this stable, high-surface-area foam network, the system establishes an efficient mass-transfer zone that maximizes contaminant exposure to the extraction and treatment processes.

Moreover, this mechanism allows the foam to serve as an effective medium for uniformly distributing chemical oxidants throughout the contaminated zone. In this study, oxidative foam was generated using a dedicated foam generator (Figure 1) with a surfactant solution containing 32 mM AOS and 1500 mM SPS. The generated oxidative foam was continuously injected into the saturated soil zone. The effective SPS concentration within the soil reactor was estimated based on the total oxidant mass delivered during the 3 h injection period at an AS flow rate of 250 mL min^–1^. Accordingly, the final SPS concentration in the reactor pore space was calculated using eq 5, yielding an available oxidant concentration of approximately 400 mM (detailed calculation in Table S3 (Supporting Information)). This concentration provided a stoichiometric basis for evaluating oxidant availability relative to the total TCE mass (6132 mg) for mineralization during both the active injection and subsequent static phases.

5 $$C_{SPS\left(reactor\right)}=\frac{C_{SPS\left(generator\right)}\times V_{liquid\left(injected\right)}}{V_{liquid\left(reactor\right)}}$$

where *C*_SPS(reactor)_ and *C*_SPS(generator)_ (mM) denote the SPS concentration in the soil reactor and foam generator, respectively, *V*_liquid(injected)_ (mL) represents the volume of liquid injected into the soil reactor at *Q*_AS_ of 250 mL min^–1^ (calculated using eq 6), and *V*_liquid(reactor)_ (mL) is the total liquid volume in the reactor after 3 h of operation.

6 $$V_{liquid\left(injected\right)}=Q_{\text{AS}}\times t\times \left(100\%-FQ\right)$$

where *t* (min) represents the operational duration of the Oxi-FEAS-SVE system and FQ (%) represents AOS foam quality (∼99.6%).^15^

HPLC/PDA analysis showed that AOS and SPS coelute between 1.2 and 2.6 min, producing overlapping peaks that prevent their independent quantification (Figure S1, Supporting Information). Because the specific chemical interactions between the AOS surfactant and SPS oxidant remain unclear, further investigation using alternative analytical methods is required. Despite this limitation, the TCE distribution observed during the 3 h active phase in the Oxi-FEAS-SVE system (Figure 2e) was highly consistent with that of the oxidant-free AOS system (Figure 2d). This similarity suggests that SPS has a negligible effect on the fundamental foam properties. For example, the AOS/SPS foam (32 mM/1700 mM) exhibited a foam quality of ∼99.6% and stability of ∼3 h, comparable to systems without SPS.^15^ Consequently, the foam maintained its effectiveness in promoting TCE dissolution and volatilization throughout the 3 h operation, even in the presence of SPS, demonstrating its suitability as a robust distribution medium for oxidative remediation.

Surfactants are essential in the Oxi-FEAS-SVE process because they enhance DNAPL mass transfer and improve foam stability. However, their interactions with persulfate should be carefully considered, as surfactants may act as competitive scavengers for persulfate-derived radicals.^23,24^ Therefore, both the surfactant type and the surfactant-to-oxidant ratio must be optimized to minimize interference with oxidation while maintaining sufficient foam performance. Luong and Liang (2025)^15^ reported that SPS reduced oxidative foam stability from 345 to 180 min for a 32 mM AOS/1700 mM SPS formulation, while the foam quality remained nearly unchanged at 99.6%. Based on this finding, the 32 mM AOS/1500 mM SPS formulation used in the present study was considered sufficient to maintain oxidative foam stability during the 3 h sparging period. This suggests that oxidative foam transport could be sustained throughout the designated operation period.

Surfactant-induced mobilization and interfacial tension reduction can enhance the transfer of DNAPL into the aqueous phase,^25^ thereby increasing contact between coinjected PS and dissolved TCE for oxidative degradation. Nevertheless, excessive DNAPL mobilization may pose a risk of uncontrolled contaminant migration. In this study, surfactant was introduced in the form of foam rather than as a direct surfactant solution, which may help limit DNAPL mobilization. In addition, air sparging during FEAS operation and the integrated SVE system were used to capture volatilized TCE and control vapor-phase migration during treatment. As reported by Shaeel Al-Thabaiti et al. (2023),^26^ surfactants may inhibit PS activation and reactive species generation through micellar interactions and radical scavenging. Therefore, maintaining an appropriate surfactant-to-oxidant ratio is critical for balancing TCE mobilization, foam stability, and oxidative degradation efficiency.

Comparison of the magnified regions in FEAS-SVE (Figure 2g) and Oxi-FEAS-SVE (Figure 2h) indicates minimal changes in TCE concentration contours. This outcome likely results from the limited reaction time between SPS and TCE in the reactor,^15^ and the relatively low operating temperature (∼25 °C), which slows persulfate thermal activation kinetics. Liang and Bruell^18^ showed that persulfate exhibits low reactivity unless activated, while thermal activation (e.g., 40 °C) produces highly reactive sulfate radicals (SO_4_^•–^, *E* ^o^ = 2.6 V), stronger than PS anion alone (*E* ^o^ = 2.01 V). Because the present study operated at 25 °C, slower reaction kinetics were expected. Accordingly, TCE degradation under ambient conditions was evaluated using the kinetic model proposed by Luong and Liang^15^ (eq 7), which was then used to estimate the reaction time required for sufficient mineralization and to design the subsequent static-period experiment.

$$-d\left[TCE\right]/dt=\left(6.90\times 10^{-5}\;{mM}^{0.2}\min^{-1}\right){\left[TCE\right]}^{0}\times {\left[S_{2}{O_{8}}^{2-}\right]}^{0.8},\left(@25\;{}^{\circ}C\right)$$ 7

In both the FEAS-SVE (Figure 2d) and Oxi-FEAS-SVE (Figure 2e) systems, soil TCE residuals decreased to 38–44%, with volatilization accounting for more than 50% of the total mass transfer (Figure 3). As summarized in Table S6 (Supporting Information), approximately 60.4 g of SPS remained in the reactor after 3 h of Oxi-FEAS-SVE operation (Experiment e), compared with a total injected amount of 64.3 g. Based on these results, a subsequent 48 h static phase was implemented to provide sufficient reaction time for TCE mineralization after active system operation. This duration was determined based on kinetic modeling of the residual oxidant capacity following the 3 h active Oxi-FEAS-SVE operation in Experiment e, considering the kinetic limitation of persulfate activation at ambient temperature. Detailed calculations supporting the selected 48 h reaction period are provided in Table S5 (Supporting Information). It is important to note that the Oxi-FEAS-SVE system operates through a two-stage treatment process. During the 3 h active operation, the primary removal mechanism is foam-enhanced dissolution and physical volatilization, driven by increased gas–liquid interfacial contact. Although chemical oxidation may also occur during this phase, the overall removal behavior is dominated by mass transfer and vapor extraction. The subsequent 48 h static phase was therefore implemented to allow sustained contact between the residual SPS-containing foam and the remaining TCE within the porous medium. This stage promotes chemical mineralization of residual TCE after active gas flow and vapor stripping have ceased. Accordingly, an additional experiment (Experiment f) was conducted to further evaluate the oxidizing capacity of SPS for degrading residual TCE within the soil matrix. The key advancement of this study is illustrated in Figure 2f,i (reduced scale). Following the 3 h active treatment and subsequent 48 h static period, soil TCE concentrations were effectively reduced to a mean of 42.9 ± 0.4 mg kg^–1^ dry soil (Experiment f, Table 1). This indicates that the static phase enabled the AOS/SPS oxidative foam to function as an oxidant delivery medium, thereby promoting the degradation of residual TCE. Concurrently, the SPS concentration in the saturated layer declined from 17-20 to 14–15 g kg^–1^ (dry soil) (Table S6, Supporting Information), indicating continued SPS decomposition. As a result, TCE mineralization increased to 37% after the 48 h static period, compared with only 4% observed after the initial 3 h of active Oxi-FEAS-SVE treatment (Figure 3). To differentiate chemical oxidation from physical removal pathways during the 48 h static phase, an additional control experiment, Experiment d, was conducted using AOS foam injection without SPS oxidant. As shown in Figure S2 (Supporting Information), the AOS-foam control exhibited negligible chloride release, indicating that measurable TCE mineralization did not occur in the absence of SPS. Moreover, comparison of the soil TCE concentration contour maps after the 3 h active phase and after the 48 h static phase showed that the residual TCE distribution remained largely unchanged, confirming that further physical removal was limited once active sparging ceased. In contrast, the Oxi-FEAS-SVE system containing SPS achieved 37% mineralization during the 48 h static phase. Therefore, this mineralization is primarily attributed to chemical oxidation resulting from sustained contact between SPS-containing foam and residual TCE within the porous medium. Although a sterilized control was not included, biodegradation was considered negligible due to the use of inert silica sand and the short experimental duration. Mineralization was quantified through stoichiometric chloride release, as detailed in Table S7 (Supporting Information). These findings demonstrate that the static phase enabled the AOS/SPS foam to function as an oxidant delivery medium, promoting the degradation of residual TCE that remained physically inaccessible during the active sparging and SVE processes.

The mass distribution results in Figure 3 indicate that volatilization was the dominant TCE removal pathway during the 3 h of active operation. While SVE alone and AS-SVE recovered only a small fraction of TCE via volatilization (<2%), the injection of AOS foam dramatically enhanced removal efficiency. In both FEAS-SVE and Oxi-FEAS-SVE systems, volatilization accounted for approximately 50–52% of the total TCE mass transfer. This substantial increase is consistent with the TCE gas-phase concentration profiles presented in Figure 4. The raw data, including standard deviations, are provided in Table S8 (Supporting Information), and this trend is further supported by the kinetic analysis of the volatilization rates.

**Figure 4:**
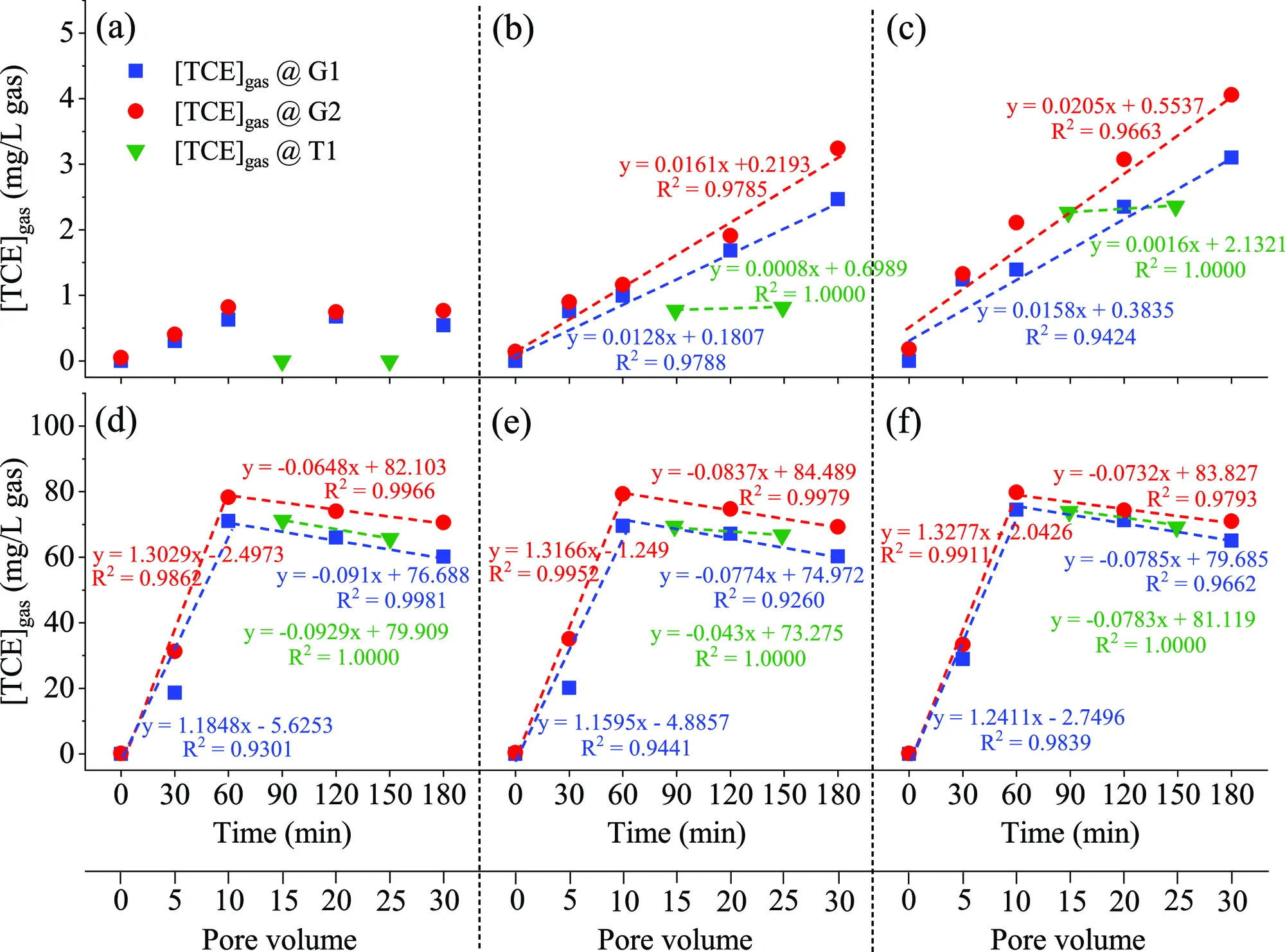
Variation of TCE concentration in the gas phase under experimental conditions: (a) control (no treatment, Experiment a); (b) SVE alone (Experiment b); (c) AS-SVE (Experiment c); (d) FEAS-SVE utilizing AOS foam (Experiment d); (e) Oxi-FEAS-SVE utilizing AOS/SPS foam after 3 h of operation (Experiment e); and (f) Oxi-FEAS-SVE utilizing AOS/SPS foam after 3 h of operation followed by 48 h of static condition (Experiment f).

In this study, pore volume (PV) is defined as the total volume of interconnected void spaces within the soil matrix. It was calculated by multiplying the total soil volume within the ROI by the effective soil porosity (*n* = 0.39) (Table S1 (Supporting Information)). Therefore, 1 PV represents the volume of air required to replace the existing soil gas within the 9 cm ROI. During the first 10 PV, linear regression of the concentration curves revealed a clear contrast among treatments. For SVE alone and AS-SVE, slopes at monitoring points G1 and G2 were very low (*r* = 0.013–0.021 mg TCE (L·min)^−1^), reflecting severe mass-transfer limitations and preferential gas channeling associated with gas-only injection, where TCE gas concentrations remained below 5 mg L^–1^ (Figure 4a–c). In contrast, FEAS-SVE and Oxi-FEAS-SVE exhibited volatilization rates approximately 73 times higher than those of SVE alone and AS-SVE (*r* = 1.160–1.328 mg TCE (L·min)^−1^). Correspondingly, gas-phase concentrations rapidly increased to peak values of ∼80 mg L^–1^ within the first 10 PV (Figure 4d–f).

This rapid volatilization highlights the superior stripping efficiency of the foam system. By mitigating preferential gas channeling, the foam distributes the stripping gas more uniformly throughout the soil matrix, increasing contact between the gas phase and residual TCE. The resulting enhancement in concentration gradients promotes mass transfer from soil zones that would otherwise remain bypassed. Notably, the nearly identical gas-phase TCE concentration profiles observed in the oxidative foam experiments (Experiments e and f) compared with the AOS foam-only system (Experiment d) indicate that the addition of SPS did not interfere with the physical foam stripping mechanism. Consequently, the system effectively recovered most of the contaminant mass through volatilization, preventing lateral dissipation and enabling efficient capture of TCE vapors prior to subsequent oxidative mineralization.

Additionally, T1 gas sampling, which represents the total TCE vapor captured by the SVE system at the reactor outlet, consistently showed lower concentrations than those measured at the internal sampling points (G1 and G2). This difference likely results from volumetric dilution and mixing as mobilized vapors migrate from the saturated zone through the unsaturated zone before reaching the effluent port (T1). The effectiveness of the exhaust gas treatment unit was further confirmed by the measurements at T2. Located downstream of the GAC column, TCE concentrations at T2 remained consistently undetectable under all experimental conditions, indicating complete adsorption of mobilized TCE vapors during the SVE process and preventing atmospheric release. The adsorption performance observed in this study is consistent with the findings of Erto et al.,^27^ who reported an adsorption capacity of approximately 150 mg TCE per gram of F400 GAC at 20 °C. In this work, approximately 50–52% of the initial TCE mass (3066–3189 mg) was volatilized and subsequently captured by 25 g of F400 GAC at 25 °C, corresponding to an experimental loading of approximately 123–128 mg TCE per gram of GAC. This value closely agrees with the reported adsorption capacity.^27,28^ The comparison confirms that the GAC column operated within its breakthrough limits and effectively handled the high-rate mass transfer generated by the oxidative foam.

Although the Oxi-FEAS-SVE system achieved high TCE removal efficiency of approximately 91% in this laboratory-scale study (Figure 3, Experiment f), several limitations should be considered when relating these results to field applications. First, the reactor was packed with homogeneous silica sand, whereas actual aquifers commonly exhibit geological heterogeneity, including variations in grain size, permeability, stratification, and preferential flow pathways. These factors may affect foam propagation, oxidant delivery, air distribution, and contaminant mass transfer. Second, the laboratory setup contained only a 5 cm saturated zone, which may have become partially or largely desaturated during air injection and vapor extraction. In contrast, field-scale DNAPL source zones may occur at greater depths within thicker and more persistent saturated intervals, where hydrostatic pressure and capillary forces could influence the foam-to-gas ratio, bubble stability, and contact between the oxidative foam and TCE. Third, although the laboratory system maintained a controlled 1:1 injection-to-extraction ratio, field-scale implementation would require careful design of injection and extraction flow rates, radius of influence, pressure distribution, and vapor capture efficiency to prevent fugitive emissions. Therefore, while the present results support the feasibility of Oxi-FEAS-SVE as an integrated remediation approach, pilot-scale studies are needed to further evaluate its performance under realistic hydrogeological conditions and to optimize operational parameters for field applications.

### 3.2 Formation of TCE Degradation Byproducts and Proposed Reaction Pathways

In the Oxi-FEAS-SVE experiments, soil samples were analyzed for potential TCE degradation intermediates after 3 h of operation followed by a 48 h static period. In this system, SPS thermally decomposes at ambient temperature (∼25 °C) to generate SO_4_^•–^ according to eq 8, although the reaction proceeds relatively slowly.^15,29^ The generated SO_4_^•–^ can further react with water molecules or OH^–^ to produce ^•^OH through radical conversion over a wide range of pH conditions (eqs 9 and 10).^30^ This conversion process releases protons (H^+^), which can result in a decrease in solution pH. Such acidification is an expected behavior associated with persulfate activation and is consistent with the oxidative pathways proposed in this study. Although thermal activation of PS is kinetically limited at ambient temperatures, previous foundational studies have verified the generation of SO_4_^•–^ and ^•^OH from PS activation within the temperature range of 10–30 °C using systematic radical quenching experiments. Liang et al.^31^ demonstrated that SO_4_^•–^ is the dominant radical species under acidic conditions, whereas relatively more ^•^OH is generated at higher pH, based on the distinct inhibition effects of *tert*-butyl alcohol (TBA) and ethanol (EtOH). In addition, Luong and Liang^15^ reported that a FEAS system achieved approximately 74% TCE mineralization when treating approximately 10 g of TCE DNAPL in 1 L of water with a PS concentration of 1700 mM at 25 ± 2 °C after 240 h of oxidation. In the present study, the substantially higher TCE removal achieved by the Oxi-FEAS-SVE system compared with the physical-only control groups, together with the detection of chlorinated degradation intermediates, is consistent with the radical-mediated TCE degradation pathways reported in the literature. Through reactions with SO_4_^•–^ and ^•^OH, TCE undergoes multiple transformation pathways, forming various intermediate products, including 1,1,2,2-tetrachloroethane (C_2_H_2_Cl_4_, TeCA), trichloromethane (CHCl_3_),^32,33^ dichloromethane (CH_2_Cl_2_),^34^ chloromethane (CH_3_Cl),^34^ and Cl^–^.^35,36^ The detection of these intermediates in the GC/MS chromatogram (Figure 5a) provides clear evidence for the stepwise degradation of TCE during SO_4_^•–^ based oxidation.

$$S_{2}{O_{8}}^{2-}\overset{\begin{array}{l} thermal\;activation\\ ambient∼99\;{}^{\circ}C \end{array}}{\to }2S{O_{4}}^{•-}$$ 8

SO4•−+H2O→SO42−+H++OH• 9

10 SO4•−+OH−→SO42−+OH•

**Figure 5:**
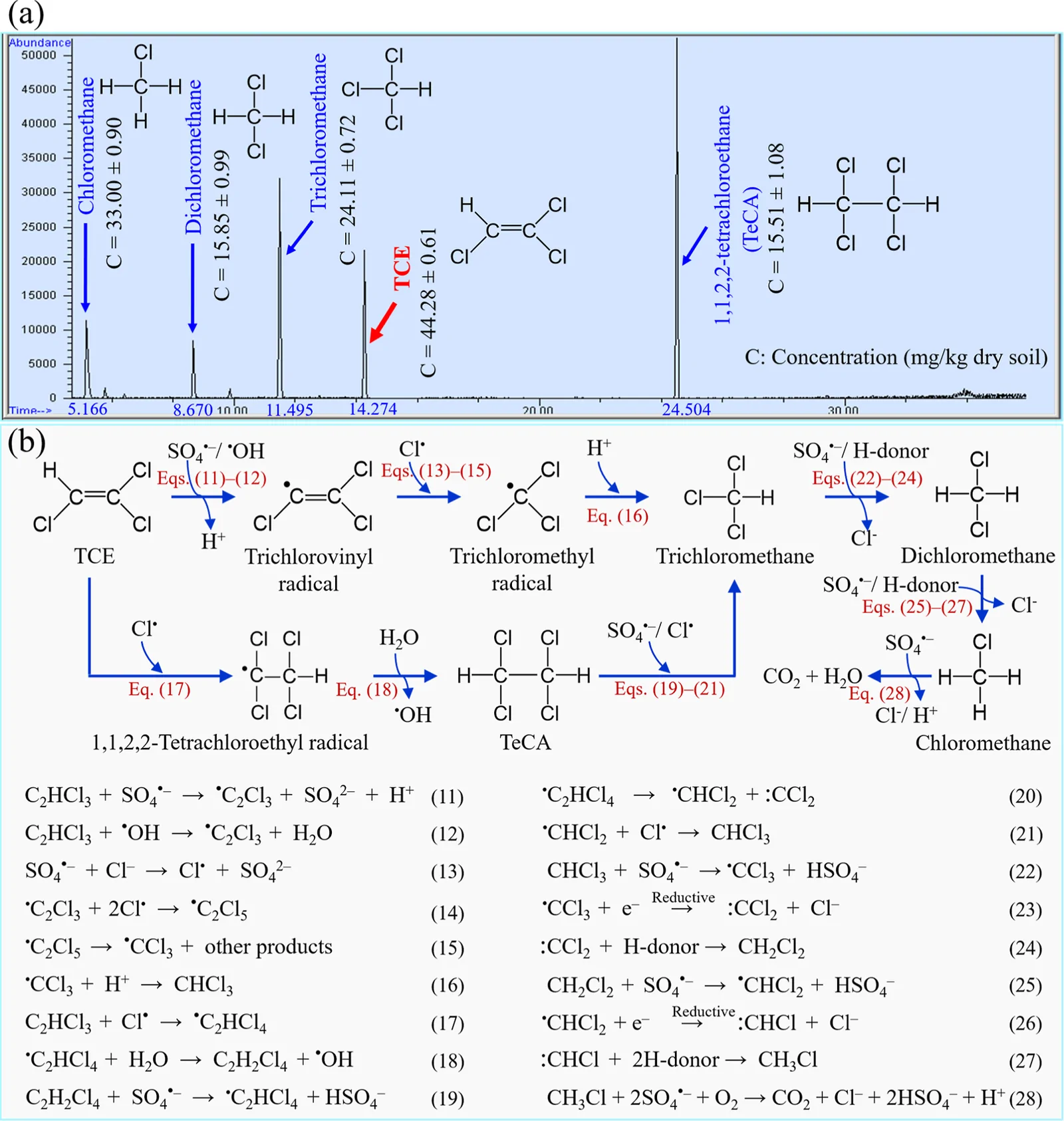
(a) GC/MS chromatogram showing TCE and its associated degradation byproducts in soil treated by Oxi-FEAS-SVE using AOS/SPS oxidative foam after 3 h of operation followed by 48 h of static incubation; (b) proposed degradation pathways of TCE.

The degradation of TCE proceeds through several reaction pathways, including radical addition, hydrogen abstraction or addition, and stepwise dechlorination, ultimately leading to mineralization,^31,37^ as illustrated in Figure 5b. The process is primarily initiated by the highly reactive SO_4_^•–^ and ^•^OH radicals. In the initial step, these radicals abstract hydrogen from TCE, forming a trichlorovinyl radical (^•^CCl=CCl_2_) (eqs 11 and 12). This intermediate is proposed to undergo further transformation through reactions with chlorine radicals (Cl^•^). A proposed step in this pathway is the generation of Cl^•^ radicals via the reaction between SO_4_^•–^ and Cl^–^ (eq 13). In the present system, the Cl^–^ ions originate from the AOS surfactant used for foam generation. Although AOS has a high purity (≥97.0%), it contains approximately 1% sodium chloride (NaCl) as an inherent component. It is suggested that this trace chloride can react with SO_4_^•–^ to produce Cl^•^ radicals, thereby potentially facilitating subsequent reactions in the TCE degradation pathway. Although the AOS surfactant contains approximately 1% NaCl as an inherent component, this trace chloride source is considered a possible initiator for Cl^•^ formation through reaction with SO_4_^•–^ (eq 13), rather than the sole chloride source sustaining the reaction pathway. During TCE degradation, dechlorination of TCE and its chlorinated intermediates can continuously release chloride ions into the aqueous phase, as described in eqs 23, 26, and 28. These released chloride ions may subsequently react with SO_4_^•–^ to regenerate Cl^•^, thereby contributing to the continuation of the radical-mediated degradation pathway. Supporting evidence is provided by the stoichiometric chloride release analysis in Table S7 (Supporting Information). The control experiments (Experiments a–d) showed no detectable chloride release, whereas the Oxi-FEAS-SVE experiments (Experiments e and f) exhibited significant chloride accumulation. This result indicates that chloride release was associated with active TCE degradation and supports the proposed role of chloride regeneration in the reaction pathway.

The trichlorovinyl radical may reacts with Cl^•^ to form a chlorinated intermediate (eq 14), which subsequently undergoes C–C bond cleavage to produce a trichloromethyl radical (^•^CCl_3_) and other fragments (eq 15). The ^•^CCl_3_ radical readily abstracts hydrogen from water or other donors, forming CHCl_3_ a relatively stable product (eq 16). Alternatively, SO_4_^•–^ or Cl^•^ may add to the C=C bond of TCE, potentially leading to bond saturation and formation of tetrachloroethane (TeCA) (eqs 17 and 18). The presence of TeCA suggests that radical addition pathways are likely significant and that further oxidation can produce additional chlorinated byproducts such as CHCl_3_ (eqs 19–21).

Trichloromethane undergoes sequential dechlorination through radical-mediated reactions, leading to the stepwise formation of CH_2_Cl_2_ (eqs 22–24) and CH_3_Cl (eqs 25–27). Both SO_4_^•–^ and ^•^OH promote hydrogen abstraction and chlorine elimination at each stage, thereby facilitating progressive dechlorination and oxidation. The hydrogen atoms required to convert the reactive carbene intermediates (:CCl_2_ and :CHCl) into stable chlorinated alkanes (eqs 24 and 27) could be supplied by the C–H bonds in the long-chain hydrocarbon tails (C14–C16) of the AOS surfactant through radical-mediated hydrogen abstraction. In addition, the reductive transformations described in eqs 23 and 26 may occur via single-electron transfer from the surfactant. During this process, it is hypothesized that AOS acts as a sacrificial electron donor; its reaction with SO_4_^•–^ may generates intermediate organic radicals (AOS^•^).^38^ These radicals may possess reducing capacity and potentially donate electrons to the more electronegative chlorinated radicals, thereby promoting their reduction and facilitating the dechlorination pathway. Consequently, it is proposed that the surfactant exhibits dual functionality: it not only serves as a physical mobility-control agent for foam formation but also may function as a chemical electron and hydrogen donor that likely sustains the reaction propagation during the 48 h static period.

Following treatment, the residual TCE concentration in the soil decreased to 44.28 mg kg^–1^ (Figure 5a), which is below the Taiwan Ministry of Environment regulatory limit of 60 mg kg^–1^,^39^ confirming that the Oxi-FEAS-SVE system can meet environmental standards. Mineralization was evaluated using a 3/1 (Cl^–^/TCE) stoichiometric balance, reflecting the release of three chlorine equivalents for each mole of TCE degraded.^18^ As shown in the inset of Figure 3, the total molar chlorine associated with the detected byproducts was calculated to be 1.99 mmol, corresponding to approximately 0.66 mmol of TCE based on the parent compound stoichiometry. The cumulative mass of these identified organic intermediates therefore represented only 1.4% of the initial TCE mass. This small fraction indicates that most of the degraded contaminant was converted into inorganic products rather than accumulating as intermediate organic pollutants. While stoichiometric chloride release analysis provides evidence of TCE dechlorination and degradation, the incomplete carbon mass balance is acknowledged as a limitation of this study. The identified organic intermediates accounted for only a small fraction of the initial TCE mass, indicating that a substantial portion of carbon was not directly quantified. This unaccounted carbon may be associated with further oxidation to CO_2_, undetected transient intermediates, or gas-phase losses during the SVE-coupled operation. Because CO_2_ was not directly captured or measured, the degree of mineralization was indirectly estimated based on chloride release stoichiometry rather than direct carbon recovery. Future studies using carbon labeling, CO_2_ capture, or gas-phase carbon analysis are recommended to establish a complete carbon mass balance and more accurately quantify mineralization efficiency. Nevertheless, the presence of residual intermediates highlights the importance of the 48 h static period. This extended reaction phase allows continued oxidation of the carbon backbone to CO_2_ and H_2_O (eq 28) and promotes the final release of inorganic chloride. The transformation of complex chlorinated intermediates into simple inorganic products therefore demonstrates that a static period is necessary to achieve near-complete mineralization. Although these finding underscore the effectiveness of the static period under controlled conditions, it is crucial to contextualize the scope of the experimental setup within the broader challenges of soil remediation. While this study demonstrated the efficacy of the Oxi-FEAS-SVE system in an inert porous medium, it is important to acknowledge that the use of silica sand served as an initial validation of the technology. Real-world subsurface conditions are significantly more complex, particularly regarding the presence of natural organic matter (NOM) and mineral heterogeneity. These factors may exert competitive oxidant demand and influence the transport and stability of the injected foam. Consequently, future research is warranted to evaluate the degradation kinetics and overall system performance in more complex, organic-matter-rich soil matrices to further bridge the gap between bench-scale findings and field-scale applications.

## 4 Conclusions

This study demonstrates that the Oxi-FEAS-SVE system is an effective alternative to conventional remediation technologies, achieving a total TCE mass removal efficiency of approximately 91%. In contrast, conventional SVE and AS-SVE showed limited performance (<2%) due to gas channeling and mass-transfer limitations. The dual-stage Oxi-FEAS-SVE approach effectively overcame these barriers through the multifunctional role of the AOS/SPS (32 mM/1500 mM) oxidative foam. During the 3 h active injection phase, the foam acted as a solubilization-enhancing agent, significantly increasing interfacial contact and facilitating 54% TCE removal through volatilization. Subsequently, during the 48 h static period, the system functioned as a chemical reservoir that sustained sulfate radical generation, enabling an additional 37% TCE mineralization. The occurrence of systematic oxidative degradation was confirmed by the detection of several chlorinated intermediates, including 1,1,2,2-tetrachloroethane, trichloromethane, dichloromethane, and chloromethane, followed by the ultimate formation of Cl^–^ ions. Beyond laboratory-scale validation, these findings provide potential implications for field application. The oxidative foam induced significant gas-flow diversion, which can mitigate aquifer heterogeneity and promote a more uniform treatment sweep compared with conventional air sparging. Moreover, the minimal residual byproduct fraction (1.4%) indicates that the integrated dissolution-oxidation strategy effectively destroys contaminants while minimizing the risk of intermediate plume migration. Although the Oxi-FEAS-SVE system demonstrates significant potential for the rapid depletion of TCE DNAPL source zones under laboratory-scale conditions, future pilot- and field-scale studies are essential to evaluate system performance under realistic site constraints, including subsurface heterogeneity, groundwater flow, and hydrostatic pressure.

## Supplementary Material



## Notes

Characteristics of the soil medium and reactor configuration (Table S1); estimated extraction flow rates as a function of treatment radius (Table S2); detailed experimental design for TCE DNAPL remediation using Oxi-FEAS-SVE (Table S3); comparison of empirical and theoretical vacuum pressures in SVE systems (Table S4); TCE and SPS concentrations in soils under varying experimental conditions (Tables S5 and S6); stoichiometric mineralization efficiency calculations based on chlorides release (Table S7); TCE concentrations in the gas phase under varying experimental conditions (Table S8); HPLC/PDA chromatograph of AOS/SPS (Figure S1); TCE mass distribution and contour map of soil TCE concentrations in FEAS-SVE experiment (Figure S2) (PDF)
