# Supplementary material for: Foam-Enhanced Air Sparging Coupled with Soil Vapor Extraction for Remediation of Trichloroethylene DNAPL-Contaminated Soil
Source: ACS Omega. 2026 Aug 29;11(36):53891–903. doi: 10.1021/acsomega.6c03103 (PMC13584519; doi:10.1021/acsomega.6c03103)
Supplement: Supplementary file 1 [file ao6c03103_si_001.pdf]

Supplementary Materials

Foam-Enhanced Air Sparging Coupled with Soil Vapor Extraction for Remediation of Trichloroethylene DNAPL-Contaminated Soil

Xuyen Thi Hong Luong, Chenju Liang*

Department of Environmental Engineering, National Chung Hsing University

145 Xingda Rd., South Dist., Taichung, 402, Taiwan

*Corresponding author. Tel.: +886-4-22856610; Fax: +886-4-22856610

Chenju Liang email: cliang@nchu.edu.tw

ORCID: orcid.org/0000-0002-5028-4668

Xuyen Thi Hong Luong email: xuyenluong@yahoo.com

ORCID: orcid.org/0009-0001-1807-8901

Contents

Table S1. Characteristics of the soil medium and specification of the soil reactor.

Table S2. Estimated extraction flow rate as a function of treatment radius and varying PVERs.

Table S3. Experimental design for remediating TCE DNAPL contamination in soil phase using Oxi-FEAS-SVE.

Table S4. Comparison of measured and theoretical vacuum pressures at varying airflow rates in SVE systems.

Table S5. TCE concentrations in soils at different sampling positions under various experimental conditions.

Table S6. SPS concentrations in the Oxi-FEAS-SVE system at different sampling locations under various experimental conditions.

Table S7. Mineralization efficiency of TCE in the Oxi-FEAS-SVE system based on chloride release.

Table S8. TCE concentrations in the gas phase at different sampling positions under various experimental conditions.

Figure S1. HPLC/PDA chromatograph of (a) AOS surfactant alone (10 mM), (b) SPS alone (5 mM), and (c) AOS/SPS (10 mM/5 mM) at a wavelength of 233 nm.

Figure S2. TCE mass distribution and contour map of soil TCE concentrations in FEAS-SVE experiment utilizing AOS foam after 3 h of operation followed by 48 h of static condition (experiment (d-48)).

Table S1. Characteristics of the soil medium and specification of the soil reactor.

Parameter		Value
**Soil medium**		
Silica sand	Particle size (mm)	0.21–0.60
	Hydraulic conductivity	0.0156 cm s^{-1}
	Porosity	0.39
Bentonite	Particle size (mm)	0.09–0.10
	Hydraulic conductivity	10^{-8} cm s^{-1}
**Soil reactor**		
Soil reactor dimension (D/H)		18/25 cm
Bentonite-covered thickness		2 cm
Unsaturated zone thickness		15 cm
Capillary rise zone thickness		3 cm
Saturated zone thickness		5 cm
Temperature		25 ± 1°C

Note: Silica sand was purchased from Yow Shiuan Co., Ltd. (Taiwan); Bentonite was purchased from First Chemical Works, Inc. (Taiwan).

Table S2. Estimated extraction flow rate as a function of treatment radius and varying PVERs.

r (cm) [1]	Q_{SVE} (cm^3/min) [2]									
	Time = 1 h					Time = 3 h				
	PVER [3]					PVER				
	1	5	10	20	30	1	5	10	20	30
0	0.0	0.0	0.0	0.0	0.0	0.0	0.0	0.0	0.0	0.0
1	0.3	1.5	3.1	6.1	9.2	0.1	0.5	1.0	2.0	3.1
2	1.2	6.1	12.2	24.5	36.7	0.4	2.0	4.1	8.2	12.2
3	2.8	13.8	27.6	55.1	82.7	0.9	4.6	9.2	18.4	27.6
4	4.9	24.5	49.0	98.0	147.0	1.6	8.2	16.3	32.7	49.0
5	7.7	38.3	76.5	153.1	229.6	2.6	12.8	25.5	51.0	76.5
6	11.0	55.1	110.2	220.4	330.6	3.7	18.4	36.7	73.5	110.2
7	15.0	75.0	150.0	300.0	450.0	5.0	25.0	50.0	100.0	150.0
8	19.6	98.0	195.9	391.9	587.8	6.5	32.7	65.3	130.6	195.9
9	24.8	124.0	248.0	496.0	743.9	8.3	41.3	82.7	165.3	248.0

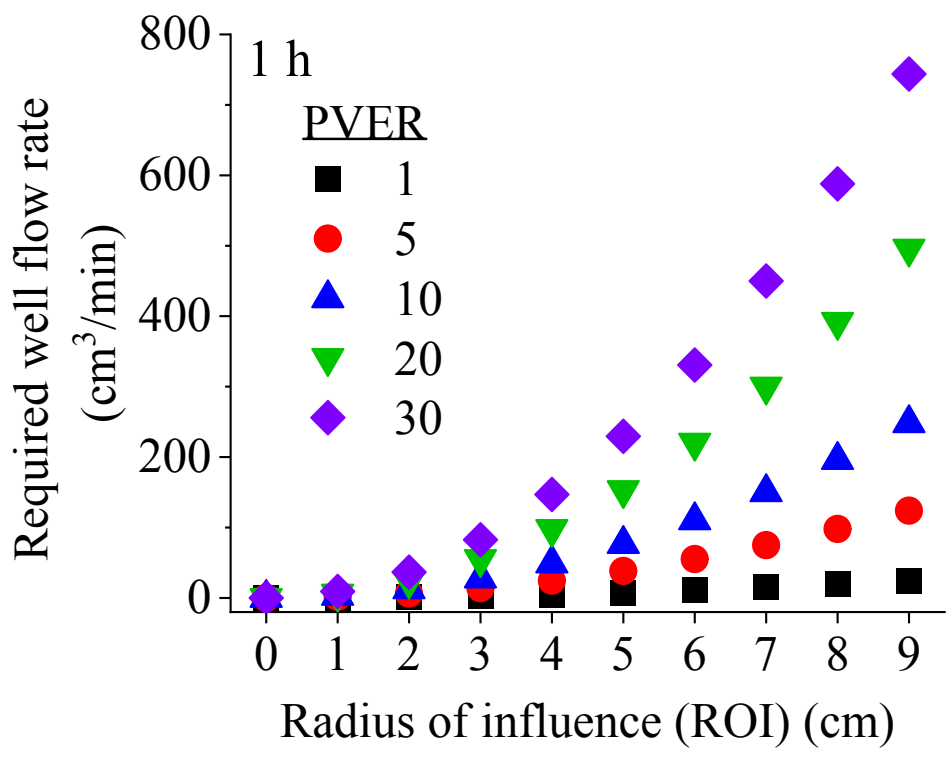

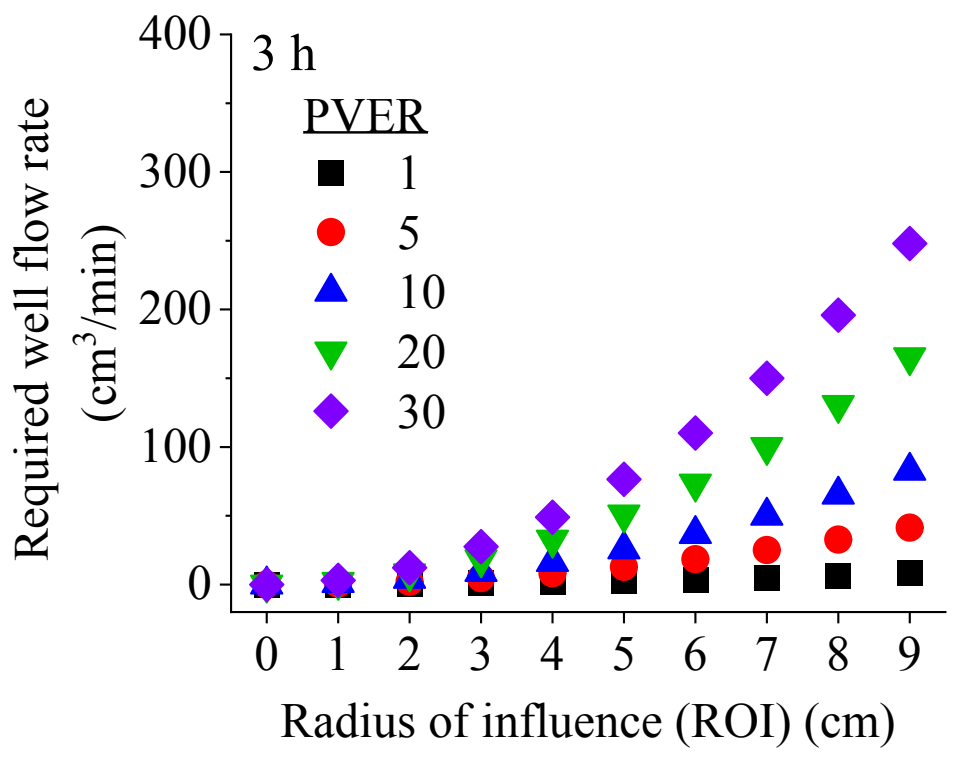

Note: [1] r (cm) is the radial distance where minimum pore volume exchange is desired. [2] Q_{SVE} (cm^3/min) = PVER × π × r^2 × h × n_e / t where [3] PVER is the pore volume exchange rate, h (cm) is the thickness of soil, n_e is the effective soil porosity (n_e = 0.39), t (min) is the operating time.

Table S3. Experimental design for remediating TCE DNAPL contamination in soil phase using Oxi-FEAS-SVE.

Group	Exp.		Q_{SVE} (mL/min)	Q_{AS} (mL/min)	[AOS] (mM) or [AOS]/[SPS] (mM/mM) in generator	Operating time	Parameters measured
Control	(a)	Static (No action)	-	-	-	3 h	TCE @ positions A1 – A4, B1 – B4, G1, G2, T1, T2, orbo tube, and GAC column (refer to Fig. 1) SPS Cl^- Byproducts
SVE alone	(b)	Extraction	250 [(1)]	-	-		
AS-SVE	(c)	N_2 injection Extraction		250 [(2)]	-		
FEAS-SVE	(d)	AOS foam			32		
	(d-48)	AOS foam				3 h for FEAS-SVE + 48 h static condition	
Oxi-FEAS-SVE	(e)	AOS/SPS foam			32/1500 [(3)]	3 h	
	(f)	AOS/SPS foam			32/1500	3 h for Oxi-FEAS-SVE + 48 h static condition	

Note: $V_{\text{pure TCE injected}}$ (mL) = 10 × $[TCE]_{max}$ vapor in the air (g/L) × V_{air} in the unsaturated soil layer (L) / TCE density (g/cm^3) = 4.2 (mL). [(1)] Q_{SVE} is calculated using equation Q = 30 × PVER × $\pi r^2 h$ × n_e / t, where PVER is pore volume exchange rate (30 times); r is radius distance (cm); h is layer of unsaturated zone (h = 15 cm); n_e is the soil porosity (n_e = 0.39); t is the operational time (min); [(2)] Assume that the ratio Q_{AS}/Q_{SVE} = 1/1. [(3)] The required C_{SPS} in foam generator (mM) was determined as follows:

- The total liquid volume in the reactor after foam injection was the sum of the initial saturated zone and the injected foam liquid: $V_{\text{Liquid (total)}} = V_{\text{Liquid (initial)}} + V_{\text{Liquid (injected)}}$ = 500 mL + 180 mL = 680 mL (0.68 L)
- The SPS concentration in the reactor was calculated based on the stoichiometric requirement for TCE mineralization (3:1 molar ratio) and a safety factor of 2:

$$C_{\text{SPS (reactor)}} = \frac{3 \times \text{Mass}_{\text{TCE}}\ (\text{mg})}{\text{MW}_{\text{TCE}}\left(\frac{\text{mg}}{\text{mmol}}\right) \times V_{\text{Liquid (total)}}\ (\text{L})} \times \text{Safety Factor}$$

$$C_{\text{SPS (reactor)}} = \frac{3 \times 6{,}132\ (mg)}{131.39\left(\frac{mg}{mmol}\right) \times 0.68\ (L)} \times 2 \approx 400\ \text{mM}$$

- The SPS concentration in the foam generator was calculated using the injection volume ratio:

$$C_{\text{SPS (generator)}} = C_{\text{SPS (reactor)}} \times \frac{V_{\text{Liquid (total)}}}{V_{\text{Liquid (injected)}}} = 400\ \text{mM} \times \frac{680\ mL}{180\ mL} \approx 1500\ \text{mM}$$

Table S4. Comparison of measured and theoretical vacuum pressures at varying airflow rates in SVE systems.

Q_{SVE} (1) (mL/min)	S_{P1} (2) (psi)	S_{P2} (3) (Monitoring) (psi)	S_{P2} (4) (Theorical calculation) (psi)
100 ± 8.66	-0.15 ± 0.01	0.00	-1.1×10^{-4}
250 ± 7.00	-0.87 ± 0.02	0.00	-2.7×10^{-4}
500 ± 5.29	-1.16 ± 0.09	0.00	-5.4×10^{-4}
800 ± 8.66	-4.93 ± 0.12	0.00	-8.6×10^{-4}
1000 ± 5.00	-7.83 ± 0.12	0.00	-10.8×10^{-4}
1200 ± 6.24	-9.86 ± 0.07	0.00	-12.9×10^{-4} (5)

Note: (1) Air vacuum pump flow rate (mL/min). (2) Vacuum of air pressure @ SVE well – P1 position (psi). (3) Vacuum of air pressure @ observation point – P2 position (psi) (refer to Fig. 1), and the vacuum pressure operates within a limited range of -0.03 psi to -14.65 psi. (4) When r ≥ 0.3R, S_{P2} is calculated by using Hantush Leaky equation:[1] $S_{P2} = \frac{3.3Q}{T} \times K_o\,[\frac{r}{B}]$ where r (ft) is the distance from center of SVE well to observation point, R (ft) is radius of influence, Q (cfm) is air vacuum flow rate, B (ft) is leaky factor, $K_o\,[\frac{r}{B}]$ is zero order modified Bassel function of the second kind,[2] T (ft^2 day^{-1}) is gas transmissivity, T = air permeability × unsaturated soil layer thickness. (5) An example calculation for Q_{SVE} = 1200 mL min^{-1} (0.0424 cfm) as shown below:

- Input parameters: r = 9 cm; R = 9 cm; B = 0.89 × R = 8.01 cm
- Bessel function: for $\frac{r}{B}$ = 1.1236; the value of $K_o\,[1.1236] \approx 0.3681$
- Gas transmissivity (T, ft^2/day) was calculated as T = k × K_A × b = 1.4365 where k is soil permeability (15.9 darcy), K_A is air permeability (0.1834 ft/day) and b is unsaturated zone thickness (b = 15 cm = 0.4921 ft)
- Pressure calculation: $S_{P2} = \frac{3.3Q}{T} \times K_o\,[\frac{r}{B}] = \frac{3.3 \times 0.0424}{1.4365} \times 0.3681 = 0.0358$ (inches-H_2O)
  = 0.00129 psi = 12.9×10^{-4} psi

Table S5. TCE concentrations in soils at different sampling positions under various experimental conditions.

Sampling position	TCE concentration (mg/kg dry soil)		
	Experiment		
	(a)	(b)	(c)
A1	6.4 ± 0.1	47.2 ± 0.5	178.1 ± 3.4
B1	6.5 ± 0.3	45.9 ± 0.4	164.8 ± 2.8
A2	9.2 ± 0.4	54.9 ± 1.9	326.9 ± 5.7
B2	9.1 ± 0.2	48.5 ± 2.6	258.2 ± 4.1
A3	65.1 ± 0.3	344.4 ± 10.2	1086.8 ± 15.3
B3	65.5 ± 0.3	304.7 ± 11.7	1065.1 ± 12.1
A4	2785.2 ± 29.8	2483.7 ± 25.6	1462.7 ± 15.8
B4	2844.5 ± 31.9	2534.0 ± 28.1	1499.3 ± 11.4
	(d)	(e)	(f)
A1	39.2 ± 0.6	28.3 ± 0.2	21.3 ± 0.4
B1	36.8 ± 0.4	25.0 ± 0.2	22.6 ± 0.5
A2	98.7 ± 0.3	80.3 ± 0.3	61.0 ± 0.3
B2	98.1 ± 0.2	80.3 ± 0.2	65.0 ± 0.8
A3	604.3 ± 4.5	471.7 ± 4.9	47.0 ± 0.5
B3	595.4 ± 3.4	488.4 ± 6.1	40.6 ± 0.3
A4	769.3 ± 5.6	721.6 ± 6.9	41.4 ± 0.2
B4	792.8 ± 6.7	739.0 ± 6.2	44.3 ±0.6

Note: (a) control experiment with static condition, (b) SVE alone, (c) AS coupled with SVE (AS-SVE), (d) Foam-enhanced AS coupled with SVE (FEAS-SVE) utilizing AOS foam, (e) Oxi-FEAS-SVE utilizing AOS/SPS foam after 3 h of operation, and (f) Oxi-FEAS-SVE utilizing AOS/SPS foam after 3 h of operation followed by a 48-h static period. The 48-h extension was determined based on the oxidation kinetics described by the following equation[3]: $-d[TCE]/dt = (6.90 \times 10^{-5}\ mM^{0.2}min^{-1})\ [TCE]^0 \times [S_2O_8^{2-}]^{0.8}$ (@25°C)
where $[S_2O_8^{2-}]$ (mM) represents the residual SPS concentration remaining in the reactor after 3-h active operation in Oxi-FEAS-SVE system (Experiment e). This was calculated from the total mass remaining (60.42 g, see Table S6 (SM)) and the reactor liquid volume (V_{liquid} = 0.68 L); The residual TCE mass in the saturated zone was calculated at ≈ 2037 mg, based on the soil concentration at positions A3, B3, A4, and B4; Applying these parameters to the kinetics model yielded an estimated extension time of ≈ 2893 min (≈ 48 h) to achieve the target mineralization.

Table S6. SPS concentrations in the Oxi-FEAS-SVE system at different sampling locations under various experimental conditions.

Sample positions	Wet soil for analysis (g)	Water content (%)	Dry soil for analysis (g)	$[SPS]_{Aq.}$ [(1)] (mg/100 mL water added)	$[SPS]_{Soil}$ [(2)] (g/kg dry soil)	SPS_T [(3)] in reactor (g)
	Experiment (e) [(4)]					
A1	30.03 ± 0.50	1.00 ± 0.15	29.73 ± 0.50	0.00 ± 0.00	0.00 ± 0.00	60.42 ± 0.62
B1	30.01 ± 1.21	1.05 ± 0.29	29.70 ± 1.20	0.00 ± 0.00	0.00 ± 0.00	
A2	30.10 ± 0.48	1.40 ± 0.20	29.68 ± 0.47	0.00 ± 0.00	0.00 ± 0.00	
B2	30.13 ± 0.86	1.57 ± 0.13	29.66 ± 0.85	0.00 ± 0.00	0.00 ± 0.00	
A3	30.04 ± 0.74	4.99 ± 0.25	28.54 ± 0.70	5.10 ± 0.09	17.86 ± 0.13	
B3	30.07 ± 0.27	5.83 ± 0.14	28.32 ± 0.26	5.21 ± 0.13	18.39 ± 0.34	
A4	30.01 ± 0.49	6.07 ± 0.56	28.19 ± 0.46	5.44 ± 0.07	19.31 ± 0.10	
B4	30.17 ± 0.95	6.01 ± 0.10	28.35 ± 0.89	5.49 ± 0.07	19.36 ± 0.42	
	Experiment (f) [(5)]					
A1	30.13 ± 1.61	1.17 ± 0.02	29.77 ± 1.59	0.00 ± 0.00	0.00 ± 0.00	46.82 ± 1.00
B1	30.21 ± 0.18	1.17 ± 0.21	29.86 ± 0.17	0.00 ± 0.00	0.00 ± 0.00	
A2	30.02 ± 1.44	1.86 ± 0.13	29.47 ± 1.42	0.00 ± 0.00	0.00 ± 0.00	
B2	30.18 ± 2.55	1.96 ± 0.37	29.58 ± 2.50	0.00 ± 0.00	0.00 ± 0.00	
A3	30.35 ± 0.52	5.18 ± 0.81	28.78 ± 0.50	4.17 ± 0.04	14.49 ± 0.11	
B3	30.11 ± 1.19	5.34 ± 0.60	28.50 ± 1.13	4.20 ± 0.04	14.73 ± 0.54	
A4	30.01 ± 1.32	6.32 ± 0.16	28.11 ± 1.24	4.18 ± 0.04	14.88 ± 0.66	
B4	30.11 ± 0.60	6.29 ± 0.32	28.21 ± 0.56	4.06 ± 0.08	14.40 ± 0.53	

Note: [(1)] SPS concentration (mg/mL) measured by adding 100 mL of water to approximately 30 g of wet soil.

[(2)] Soil SPS concentration (g/kg dry soil) calculated as $[SPS]_{Aq.}$ (g/mL) × 100 mL water added / Weight of dry soil (kg).

[(3)] Total mass of SPS in the reactor (g) calculated as $[SPS]_{Soil}$ (g/kg dry soil) × Weight of dry soil in the reactor (kg). The theoretical total mass of SPS (g) injected in the reactor after 3 h of operation is calculated using the following equation: Theoretical SPS_T (g) = C_{SPS} (M) in the generator × V_{Liquid} injected into reactor (L) × MW_{SPS} (g/mol) = 1.5 (M) × 0.18 (L) × 238 (g/mol) = 64.26 (g), where V_{Liquid} injected into reactor (L) = Q_{AS} (mL/min) × Time (min) × (100 - Foam quality (%)) / 1000 (mL/L) = 250 (mL/min) × 3 (h) × 60 (min/h) × (100 – 99.6)% / 1000 (mL/L) = 0.18 (L).

[(4)] Oxi-FEAS-SVE utilizing AOS/SPS foam after 3 h of operation.

[(5)] Oxi-FEAS-SVE utilizing AOS/SPS foam after 3 h of operation and 48 h of static condition.

Table S7. Mineralization efficiency of TCE in the Oxi-FEAS-SVE system based on chloride release.

Sample positions	$[Cl^-]$ (1) (mg/0.1 L water added)	Dry soil weight (2) (m_{dry}, g)	$[TCE]_{degraded}$ (3) (C_{deg}, mg/kg dry soil)	Degraded TCE (4) (M_{deg}, mg)	Mineralization (5) (%)
	Experiment (e) (6)				
A1	0.00 ± 0.00	29.70 ± 0.57	0.00 ± 0.00	0.00 ± 0.00	4.18 ± 0.03
B1	0.00 ± 0.00	29.69 ± 0.52	0.00 ± 0.00	0.00 ± 0.00	
A2	0.00 ± 0.00	29.58 ± 0.42	0.00 ± 0.00	0.00 ± 0.00	
B2	0.00 ± 0.00	29.53 ± 0.31	0.00 ± 0.00	0.00 ± 0.00	
A3	19.65 ± 0.36	28.50 ± 0.39	85.13 ± 1.75	51.08 ± 1.05	
B3	20.78 ± 0.27	28.25 ± 0.69	90.89 ± 3.25	54.54 ± 1.95	
A4	17.30 ± 0.12	28.18 ± 0.48	75.82 ± 1.59	75.82 ± 1.59	
B4	17.06 ± 0.12	28.20 ± 0.28	74.70 ± 1.20	74.70 ± 1.20	
	Experiment (f) (7)				
A1	0.00 ± 0.00	29.65 ± 0.60	0.00 ± 0.00	0.00 ± 0.00	37.00 ± 0.67
B1	0.00 ± 0.00	29.65 ± 1.29	0.00 ± 0.00	0.00 ± 0.00	
A2	0.00 ± 0.00	29.44 ± 1.80	0.00 ± 0.00	0.00 ± 0.00	
B2	0.00 ± 0.00	29.41 ± 0.75	0.00 ± 0.00	0.00 ± 0.00	
A3	119.26 ± 0.83	28.45 ± 1.03	518.16 ± 20.63	310.90 ± 12.38	
B3	128.79 ± 0.90	28.40 ± 0.51	560.00 ± 6.95	336.00 ± 4.17	
A4	186.53 ± 1.37	28.10 ± 0.56	819.70 ± 14.43	819.70 ± 14.43	
B4	182.71 ± 2.56	28.11 ± 0.05	802.47 ± 10.78	802.47 ± 10.78	

Note: (1) Cl^- concentration (mg/L) was measured by adding 100 mL of water to approximately 30 g of wet soil.

(2) Dry soil weight was calculated as: m_{dry} (g) = wet soil weight (30 g) × (100% – water content %)

(3) Degraded TCE concentration was derived from stoichiometric chloride release:

$$C_{deg} = \frac{[Cl^-] \times V_{water}}{MW_{Cl} \times 3} \times \frac{MW_{TCE}}{m_{dry}}$$

where V_{water} is 0.1 L, MW_{Cl} is the molar mass of chlorine (35.45 mg/mmol), and MW_{TCE} is the molar mass of TCE (131.39 mg/mmol). Calculation assume a 3:1 (Cl^-/TCE) stoichiometric balance.

(4) The total mass of degraded TCE (M_{deg}, mg) per layer was calculated as: $M_{deg} = C_{deg} \times m_{layer}$ where m_{layer} is the total dry weight of silica sand in each layer (kg).

(5) Mineralization efficiency (%) was determined by: Mineralization (%) = $\frac{M_{deg}}{\text{Initial TCE mass}} \times 100\%$

(6) Oxi-FEAS-SVE utilizing AOS/SPS foam after 3 h of operation.

(7) Oxi-FEAS-SVE utilizing AOS/SPS foam after 3 h of operation and 48 h of static condition.

Table S8. TCE concentrations in the gas phase at different sampling positions under various experimental conditions.

Time (min)	TCE conc. (mg/L gas) in the gas phase at positions G1, G2, and T1					
	Experiment (a)			Experiment (b)		
	G1	G2	T1	G1	G2	T1
0	0.00 ± 0.00	0.05 ± 0.01	-	0.00 ± 0.00	0.15 ± 0.01	-
30	0.30 ± 0.01	0.41 ± 0.02	-	0.76 ± 0.02	0.90 ± 0.05	-
60	0.63 ± 0.02	0.82 ± 0.07	-	1.00 ± 0.05	1.16 ± 0.02	-
90	-	-	0.00 ± 0.00	-	-	0.77 ± 0.06
120	0.68 ± 0.07	0.75 ± 0.06	-	1.69 ± 0.12	1.91 ± 0.13	-
150	-	-	0.00 ± 0.00	-	-	0.82 ± 0.07
180	0.54 ± 0.01	0.77 ± 0.07	-	2.47 ± 0.15	3.24 ± 0.13	-
	Experiment (c)			Experiment (d)		
	G1	G2	T1	G1	G2	T1
0	0.00 ± 0.00	0.18 ± 0.01	-	0.00 ± 0.00	0.17 ± 0.03	-
30	1.24 ± 0.03	1.33 ± 0.00	-	18.67 ± 0.88	31.24 ± 0.65	-
60	1.39 ± 0.11	2.11 ± 0.12	-	71.09 ± 0.58	78.35 ± 0.98	-
90	-	-	2.27 ± 0.11	-	-	71.55 ± 0.31
120	2.35 ± 0.05	3.07 ± 0.12	-	66.04 ± 0.94	74.07 ± 1.08	-
150	-	-	2.37 ± 0.14	-	-	65.97 ± 0.10
180	3.10 ± 0.11	4.06 ± 0.15	-	60.17 ± 0.12	70.58 ± 0.99	-
	Experiment (e)			Experiment (f)		
	G1	G2	T1	G1	G2	T1
0	0.00 ± 0.00	0.34 ± 0.01	-	0.00 ± 0.00	0.14 ± 0.01	-
30	20.13 ± 0.18	35.07 ± 0.88	-	28.98 ± 0.89	33.42 ± 1.29	-
60	69.57 ± 0.56	79.33 ± 1.03	-	74.47 ± 1.00	79.81 ± 1.19	-
90	-	-	69.41 ± 1.05	-	-	74.07 ± 1.18
120	67.20 ± 0.07	74.71 ± 1.08	-	71.28 ± 0.97	74.31 ± 1.16	-
150	-	-	66.83 ± 0.20	-	-	69.37± 1.02
180	60.28 ± 0.02	69.29 ± 1.00	-	65.04 ± 0.96	71.03 ± 0.96	-

Note: (a) control experiment with static condition, (b) SVE alone, (c) AS coupled with SVE (AS-SVE), (d) Foam-enhanced AS coupled with SVE (FEAS-SVE) utilizing AOS foam, (e) Oxi-FEAS-SVE utilizing AOS/SPS foam after 3 h of operation, and (f) Oxi-FEAS-SVE utilizing AOS/SPS foam after 3 h of operation followed by a 48-h static period. "-" represents "not measured".

References

(1) Johnson, P.C.; Stanley, C.; Kemblowski, M.W.; Byers, D.; Colthart, J. A practical approach to the design, operation, and monitoring of in situ soil-venting systems. *Ground Water Monit. Remediat.* **1990**, *10*, 159.

(2) Fetter, C.W. *Applied Hydrogeology*; Merril Publishing Co., Upper Saddle River, New Jersey, USA, **1980**.

(3) Luong, X.T.H.; Liang, C. Comparative assessment of regular and persulfate oxidative foams in air sparging for trichloroethylene dense nonaqueous phase liquid remediation. *ACS Environ. Au* **2025**, *5*, 468.

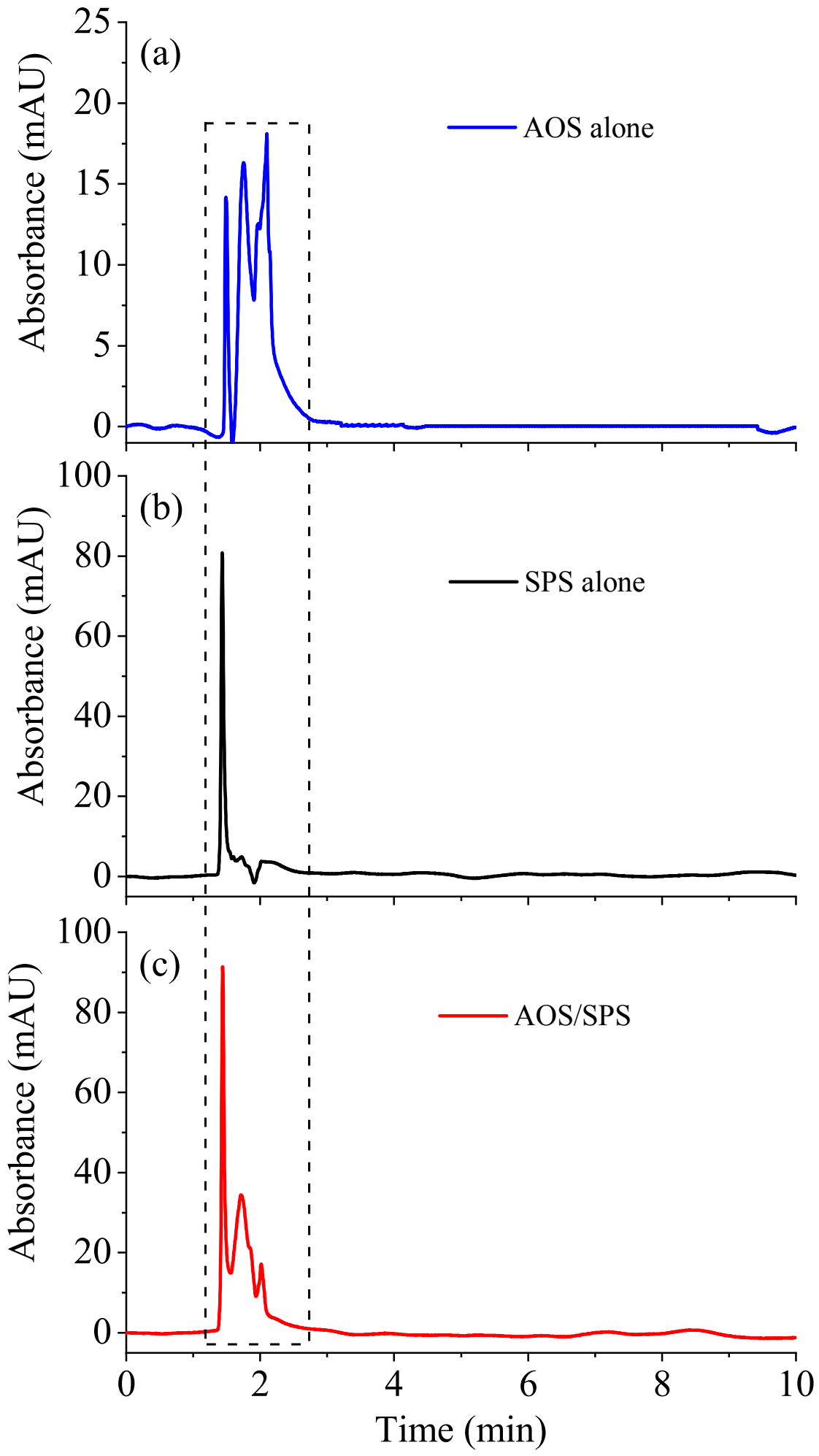

Figure S1. HPLC/PDA chromatograph of (a) AOS surfactant alone (10 mM), (b) SPS alone (5 mM), and (c) AOS/SPS (10 mM/5 mM) at a wavelength of 233 nm.

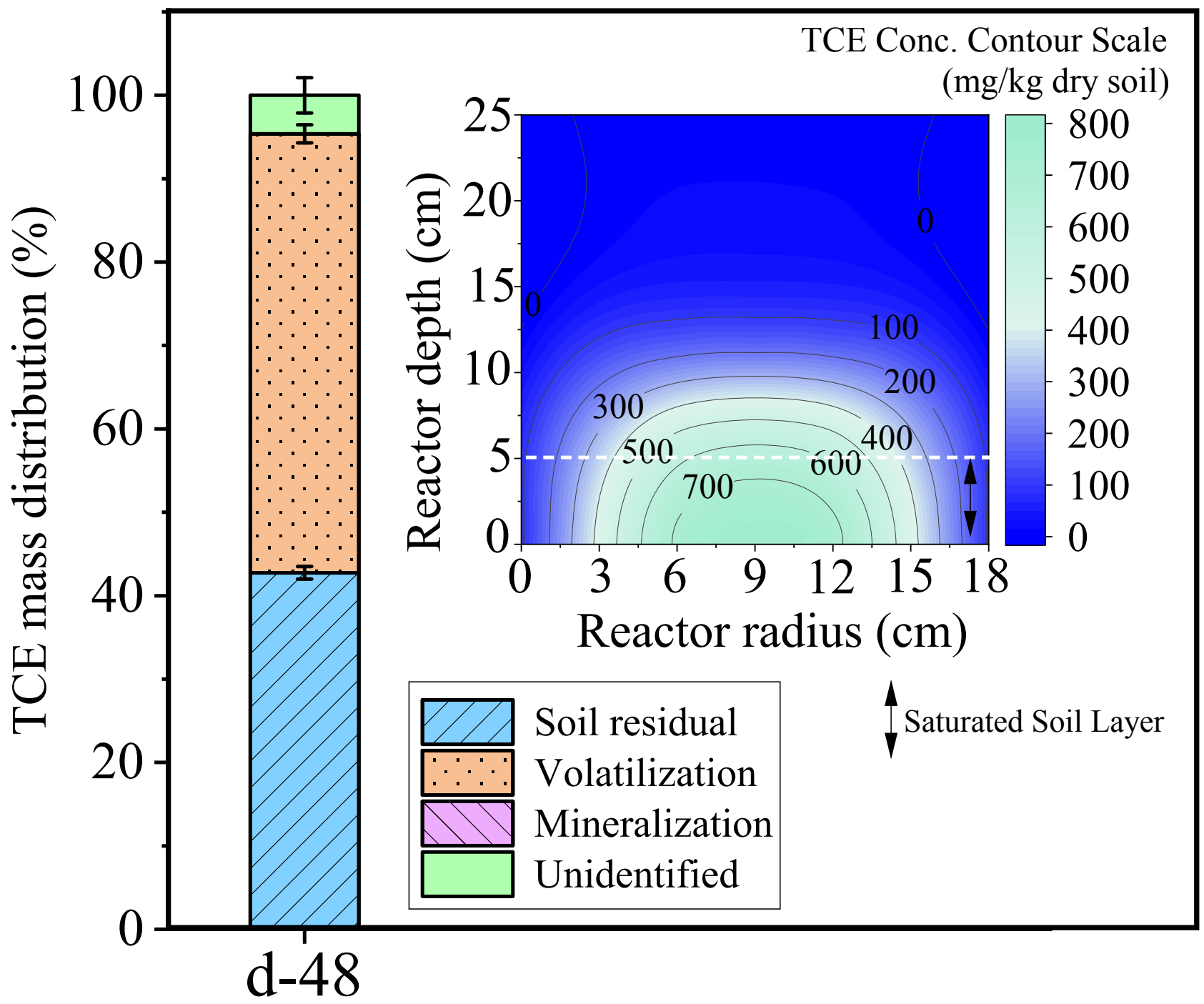

Experiment: 3 h of operation + 48 h of static condition

Figure S2. TCE mass distribution and contour map of soil TCE concentrations in FEAS-SVE experiment utilizing AOS foam after 3 h of operation followed by 48 h of static condition (experiment (d-48)).
